# Characterization of the Inflammatory Response Evoked by Bacterial Membrane Vesicles in Intestinal Cells Reveals an RIPK2-Dependent Activation by Enterotoxigenic Escherichia coli Vesicles

**DOI:** 10.1128/spectrum.01115-23

**Published:** 2023-06-12

**Authors:** Himadri B. Thapa, Paul Kohl, Franz G. Zingl, Dominik Fleischhacker, Heimo Wolinski, Thomas A. Kufer, Stefan Schild

**Affiliations:** a Institute of Molecular Biosciences, University of Graz, Graz, Austria; b Field of Excellence Biohealth, University of Graz, Graz, Austria; c Department of Immunology, Institute of Nutritional Medicine, University of Hohenheim, Stuttgart, Germany; d BioTechMed-Graz, Graz, Austria; Ludwig-Maximilians-Universitat Munchen Pettenkofer Institute

**Keywords:** bacterial membrane vesicles, outer membrane vesicles, ETEC, cytokine, intestinal epithelial cell, HT-29, porin, LPS, RIPK2, caspase, IL-8, inflammatory response, OMV, cytokines, interleukins, lipopolysaccharide, porins

## Abstract

Although the immunomodulatory potency of bacterial membrane vesicles (MVs) is widely acknowledged, their interactions with host cells and the underlying signaling pathways have not been well studied. Herein, we provide a comparative analysis of the proinflammatory cytokine profile secreted by human intestinal epithelial cells exposed to MVs derived from 32 gut bacteria. In general, outer membrane vesicles (OMVs) from Gram-negative bacteria induced a stronger proinflammatory response than MVs from Gram-positive bacteria. However, the quality and quantity of cytokine induction varied between MVs from different species, highlighting their unique immunomodulatory properties. OMVs from enterotoxigenic Escherichia coli (ETEC) were among those showing the strongest proinflammatory potency. In depth analyses revealed that the immunomodulatory activity of ETEC OMVs relies on a so far unprecedented two-step mechanism, including their internalization into host cells followed by intracellular recognition. First, OMVs are efficiently taken up by intestinal epithelial cells, which mainly depends on caveolin-mediated endocytosis as well as the presence of the outer membrane porins OmpA and OmpF on the MVs. Second, lipopolysaccharide (LPS) delivered by OMVs is intracellularly recognized by novel caspase- and RIPK2-dependent pathways. This recognition likely occurs via detection of the lipid A moiety as ETEC OMVs with underacylated LPS exhibited reduced proinflammatory potency but similar uptake dynamics compared to OMVs derived from wild-type (WT) ETEC. Intracellular recognition of ETEC OMVs in intestinal epithelial cells is pivotal for the proinflammatory response as inhibition of OMV uptake also abolished cytokine induction. The study signifies the importance of OMV internalization by host cells to exercise their immunomodulatory activities.

**IMPORTANCE** The release of membrane vesicles from the bacterial cell surface is highly conserved among most bacterial species, including outer membrane vesicles (OMVs) from Gram-negative bacteria as well as vesicles liberated from the cytoplasmic membrane of Gram-positive bacteria. It is becoming increasingly evident that these multifactorial spheres, carrying membranous, periplasmic, and even cytosolic content, contribute to intra- and interspecies communication. In particular, gut microbiota and the host engage in a myriad of immunogenic and metabolic interactions. This study highlights the individual immunomodulatory activities of bacterial membrane vesicles from different enteric species and provides new mechanistic insights into the recognition of ETEC OMVs by human intestinal epithelial cells.

## INTRODUCTION

Production and release of spherical, membranous vesicles are highly conserved among all bacterial species. Recent data show that they are an integral part of prokaryotic biology, delivering bioactive effector molecules to other bacteria and host cells (1). The biological cargo is highly diverse and complex as membrane vesicles (MVs) are liberated from Gram-negative and -positive bacterial donors, including pathogens and pathobionts as well as probiotic isolates.

One bacterial species likely liberates different MV types with dissimilar composition as different routes of vesicle release based on blebbing and cell lysis events have been reported (1). In general, these multifactorial spheres range in diameter from 10 to 300 nm and consist mainly of microbial surface components, such as phospholipids, (lipo)proteins, membrane proteins, peptidoglycan, lipopolysaccharides (LPS), or (lipo)teichoic acids. MVs can also contain cytoplasmic and periplasmic components, such as proteins, signaling molecules, and nucleic acids, which are trapped in the lumen of MVs during the vesiculation process. MVs can act as delivery vehicles for virulence factors, immunomodulatory compounds, enzymes, and DNA to enable transfer of genes or signaling molecules for intra- and interspecies communication (2, 3). Notably, MVs represent the only vehicle for the delivery of hydrophobic compounds to host cells and are therefore also considered type 0 secretion systems (4). Based on the cultivation and isolation protocols used in this study, the focus lies on blebbing-type MVs, which mainly comprise outer membrane vesicles (OMVs) released from Gram-negative bacteria and vesicles liberated from the cytoplasmic membrane of Gram-positive bacteria.

The human gastrointestinal tract is a key organ for bacterium-host communication. Despite its function as a digestive apparatus, the human gastrointestinal tract is home to a highly complex microbial community (termed the gut microbiota) of more than 100 trillion members representing a habitat with one of the highest microbial densities on Earth (5, 6). It is widely accepted that gut microbiota and the host engage various immunogenic and metabolic interactions, which impact many systemic processes such as accessibility of nutrients, complementary metabolic pathways, enteric protection, the immune response, epithelial barrier function, and even behavior. The dynamic cross talk between gut microbiota and host cells is a fundamental feature of intestinal homeostasis, but it can also trigger diseases.

It is becoming increasingly evident that bacterial MVs are players for this communication. Recent data indicate that bacterial vesiculation can be regulated and is enhanced by the host environment: i.e., envelope stress, iron limitation, and enteric viruses (7–9). Thus, epithelial cells of the gastrointestinal tract are constantly exposed to MV doses most likely higher than previously reported from *in vitro* conditions. Notably, the thick mucus layer is considered to generally reduce direct contact between gut bacteria and the intestinal epithelium, but can be passed by MVs. Thus, MVs can provide at least one communication route between microbes and host cells (10, 11). The best-studied examples include MVs from intestinal pathogens, which allow efficient delivery of virulence factors to host cells, facilitating pathogenesis. Among the first reported MV-associated virulence factors is the heat-labile enterotoxin (LT) from enterotoxigenic Escherichia coli (ETEC), which is mainly responsible for diarrheal symptoms. In detail, LT deregulates the cellular adenylate cyclase, causing a rise in cAMP levels in intestinal cells, thereby resulting in the efflux of water and electrolytes into the intestinal lumen. Moreover, LT triggers a cAMP-dependent stimulation of the Ras-like GTPase Rap1, activating the proinflammatory NF-κB signaling pathway (12, 13). About 15% of the cholera toxin (CT) of Vibrio cholerae, which is highly homologous to LT, is also associated with MVs. In contrast to CT exported via the type 2 secretion system, the MV-associated CT is fairly protected from proteolytic degradation and can enter the intestinal epithelial cells in a GM1-independent manner, broadening its target cell specificity (14–16).

Aside from virulence factor delivery, bacterial MVs are generally considered to have immunomodulatory activities as they are recognized by host cells and even internalized by nonprofessional phagocytes, such as epithelial and endothelial cells (14–16). Several mechanisms of host cell entry have been described. For example, clathrin-dependent endocytosis mediates uptake of Pseudomonas aeruginosa OMVs in lung cells, V. cholerae OMVs are predominantly internalized by intestinal cells via caveolin-mediated endocytosis, and OMVs from Aggregatibacter actinomycetemcomitans have been reported to fuse with lipid rafts the plasma membrane of HeLa cells and fibroblasts (17–19). A lipid raft-dependent uptake was also suggested for Staphylococcus aureus MVs (20). Composition of MVs may also alter uptake pathways. In the absence of VacA cytotoxin OMVs of Helicobacter pylori are mainly taken up via clathrin-dependent endocytosis, whereas VacA-carrying OMVs can utilize alternative pathways (21). Thus, diverse internalization pathways can mediate host cell entry of MVs, depending on the MV type, origin, and composition. Microbial presence and activity are generally sensed via microbial-associated molecular patterns (MAMPS), which are detected by host cell sensors also known as pattern recognition receptors (PRRs). Notably, bacterial MVs can contain various MAMPS highlighting MVs as key factors with immunomodulatory and immunostimulatory potential (22, 23). Interaction with PRRs is emerging to be complex and can involve different classes PRRs ranging from membrane-bound receptors, such as Toll-like receptors (TLRs), to cytoplasmic types, such as nucleotide-binding oligomerization domain (NOD)-containing proteins, cysteinyl-aspartate-specific protease (caspases), and intracellular TLRs.

The proinflammatory potency of bacterial MVs on diverse host cells has been shown by several studies. For example, interleukin-8 (IL-8) production is induced in stomach tissue exposed to MVs from Helicobacter pylori as well as in bronchial epithelial cells upon addition of MVs from Pseudomonas aeruginosa (24, 25). However, the details of host cell responses toward MV exposure are far from being understood, exemplified by the induction of multiple cytokines and chemokines with diverse functions (i.e., IL-6, IL-7, IL-8, IL-13, granulocyte colony-stimulating factor [G-CSF], monocyte chemoattractant protein 1 [MCP-1], and gamma interferon [IFN-γ]) in human alveolar epithelial cells stimulated with MVs from Legionella pneumophila (26). Notably, immunomodulation via MVs is not limited to induction of proinflammatory cytokine responses. For example, MVs derived from Bacteroides fragilis, Akkermanisia mucinophila, and *Lactobacillus* spp. have been associated with beneficial activity reducing proinflammatory responses (27–30).

Despite general awareness that MVs have immunomodulatory activity resulting in beneficial or adverse effects for the host, the pathways underlying these interactions remain largely elusive. Here, we provide a comprehensive comparative study that reports the cytokine induction profiles of intestinal epithelial cells upon exposure to MVs from diverse gut bacteria. Our data indicate that MVs of different bacterial species exhibit unique immunomodulatory properties in terms of the quality and quantity of cytokine induction. Focusing on OMVs from ETEC, representing strong proinflammatory efficacy, immunomodulatory activity was shown to depend on a two-factor mechanism comprising a porin-dependent internalization, followed by activation of intracellular signaling cascades mediated by OMV-associated LPS. These findings not only highlight the differences in the individual inflammatory capacity of MVs, but also provide new mechanistic insights into the recognition of MVs by intestinal epithelial cells.

## RESULTS

### MVs derived from gut bacteria induce differential cytokine responses in intestinal epithelial cells.

No comprehensive comparative analyses on cytokine responses in intestinal epithelial cells upon exposure to MVs derived from gut bacteria have been conducted so far. As a first assessment, we detected a panel of cytokines by Luminex analyses from three intestinal epithelial cell lines upon exposure to nine bacterial MVs (see Fig. S1 in the supplemental material). To ensure adequate diversity, the study comprised three well-established and frequently used human intestinal cell lines, HT-29, HT-29 MTX, and Caco-2, in combination with MVs from eight Gram-negative donors and one Gram-positive bacterial donor covering pathogens and pathobionts as well as probiotic isolates. These bacterial strains were selected based on simple laboratory cultivation as well as reproducible MV quality and quantity. Mock-treated epithelial cells served as nonstimulated background controls. Analyses focused on 23 inflammatory cytokines, which have been reported to be expressed in at least one of the intestinal cells used and/or elevated in inflammatory diseases of the intestinal tract. An MV dose of 100 ng/mL was chosen for these assays as previous reports successfully used this MV concentration to induce immune responses in host cells (31–34). The results, provided as a heat map in Fig. S1, revealed several general tendencies. (i) Some cytokines, such as CCL20/MIP-3α, CXCL8/IL-8, and CXCL1/GROα show a robust induction upon exposure to the majority of MVs, while others were not induced by addition of any MV tested. (ii) Different MVs elicited a distinctive cytokine response, indicated by an individual induction profile of the diverse cytokines as well as the induction level. (iii) HT-29 and HT-29 MTX generally showed a stronger cytokine response than Caco-2 cells.

Regarding the intestinal cells, we focused on HT-29 cells for further analyses as they exhibited a slightly better growth dynamic than HT-29 MTX cells. To validate the MV dose used in the assays, we assessed the proinflammatory IL-8 response as well as cell viability evoked by different doses of MVs from seven Gram-negative and three Gram-positive bacterial donors (Fig. S2). IL-8 was chosen as it showed the most robust MV-dependent induction in the Luminex analyses (Fig. S1). The results demonstrate that concentrations of 10 ng/mL do not elicit a response for the tested MVs, while 100 ng/mL showed a robust IL-8 induction for several MV candidates (Fig. S2A). None of the tested MVs significantly reduced cell viability at a dose of 100 ng/mL (Fig. S2B). In most cases, higher MV concentrations do not automatically yield substantially higher cytokine levels. Notable exceptions are MVs derived from Enterococcus faecalis and Lactobacillus acidophilus, which elicit almost no response with 100 ng/mL but elevated IL-8 levels at MV doses of 1 and/or 10 μg/mL, respectively (Fig. S2A). However, this is paralleled by a significant loss of cell viability (Fig. S2B). Several other MV species also significantly impair host cell viability at doses of 1 μg/mL and, in particular, 10 μg/mL. Although different MV doses might reveal different cytokine responses, in our view the chosen MV concentration of 100 ng/mL seems appropriate for the comparative analysis approach provided herein.

**FIG 1:**
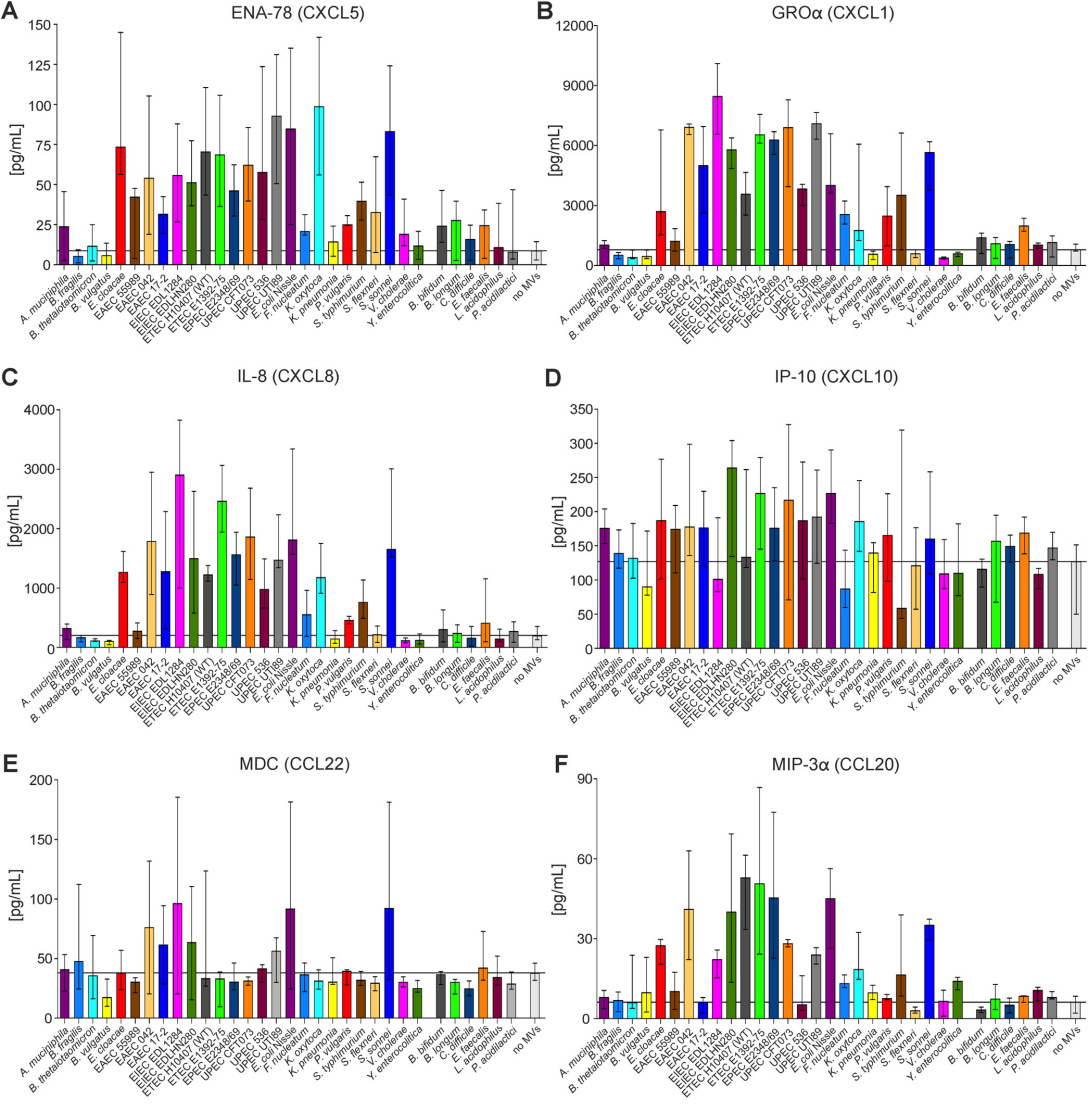
Proinflammatory cytokine response to MVs derived from diverse intestinal bacteria in human intestinal cells. ENA-78/CXCL5 (A), GRO-alpha/CXCL1 (B), IL-8/CXCL8 (C), IP-10/CXCL10 (D), MDC/CCL22 (E) and MIP-3alpha/CCL20 (F) were detected, and levels were quantified by ELISA in supernatants of HT-29 intestinal cells incubated for 16 h with equal amounts of MVs derived from the bacterial species indicated on the *x* axis, respectively. MVs are sorted alphabetically by their donor species separated by Gram-negative (left) and Gram-positive (right) bacteria. Incubation with saline served as a negative control (no MVs [far right]). The corresponding basal level of each cytokine/chemokine produced by HT-29 is given by the median value of the control (no MVs) and highlighted with a horizontal black line. Data are indicated as the median ± interquartile range. (A) *n* = 4 for B. thetaiotaomicron, F. nucleatum and Y. enterocolitica; *n* = 11 for E. cloacae and no MVs, *n* = 7 for enteroaggregative E. coli (EAEC) 55989, EPEC E2348/69, and L. acidophilus; *n* = 8 for EAEC 042, EAEC 17-2, enteroinvasive E. coli (EIEC) EDL 1284, EIEC HN280, ETEC 1392-75, uropathogenic E. coli (UPEC) CFT073, K. oxytoca, S. flexneri, and Pediococcus acidilactici; *n* = 10 for ETEC 10407 (WT) and E. coli Nissle; *n* = 5 for P. vulgaris; *n* = 9 for Shigella sonnei and V. cholerae; *n* = 6 for all other data sets. (B) *n* = 8 for B. fragilis, EAEC 55989, EAEC 042, EAEC 17-2, EIEC EDL 1284, EIEC HN280, ETEC 1392-75, EPEC E2348/69, P. vulgaris, *S.* Typhimurium, and Y. enterocolitica; *n* = 12 for Bacteroides thetaiotaomicron, Bacteroides vulgatus, E. cloacae, UPEC CFT073, E. coli Nissle, and K. oxytoca; *n* = 18 for ETEC 10407 (WT); *n* = 9 for UPEC 536; *n* = 14 for no MVs; *n* = 10 for all other data sets. (C) *n* = 12 for E. cloacae, K. oxytoca, S. sonnei, and L. acidophilus; *n* = 47 for ETEC 10407 (WT); *n* = 16 for UPEC CFT073 and V. cholerae; *n* = 4 for UPEC UTI89; *n* = 14 for E. coli Nissle; *n* = 28 for no MVs; *n* = 8 for all other data sets; (D) *n* = 6 for B. fragilis, UPEC CFT07, UPEC 536, K. pneumoniae, P. vulgaris, V. cholerae, and Y. enterocolitica; *n* = 11 for B. vulgatus; *n* = 10 for EIEC EDL 1284; *n* = 12 for ETEC 10407 (WT) and no MVs; *n* = 4 for F. nucleatum; *n* = 7 for L. acidophilus; *n* = 8 for all other data sets. (E) *n* = 12 for B. thetaiotaomicron, V. cholerae, *P. acidilactici*, and no MVs; *n* = 14 for ETEC 10407 (WT); *n* = 10 for E. cloacae and K. oxytoca; *n* = 8 for all other data sets. (F) *n* = 4 for EIEC EDL 1284; *n* = 16 for ETEC 10407 (WT) and V. cholerae; *n* = 12 for E. coli Nissle; *n* = 14 for no MVs; *n* = 8 for all other data sets.

For further analyses, we focused on selected six cytokines that showed a robust (GROα, IL-8, and MIP-3α) or moderate (CXCL5/ENA-78, CXCL10/IP-10, and CCL20/MDC) MV-dependent induction to MVs for extended analyses using MVs derived from 26 Gram-negative and 6 Gram-positive intestinal bacterial strains (Fig. 1). To compensate for effects from individual MV preparations or cell culture batches, at least two independent MV preparations were tested at least twice in independent cell culture assays. Concordant with the Luminex analyses, highly diverse immunomodulatory potencies of the individual MVs were obtained. In general, MVs from Gram-negative bacteria demonstrated stronger potency for cytokine induction than MVs from Gram-positive bacteria. Among the latter, a notable exception were MVs from Enterococcus faecalis, which reproducibly triggered secretion of GROα and IP-10, while all other MVs from Gram-positive bacteria had no relevant effect on any cytokine release. The strongest proinflammatory efficacy was observed for MVs derived from Gram-negative bacteria, such as Enterobacter cloacae, Klebsiella oxytoca, Shigella sonnei, and Salmonella enterica serovar Typhimurium as well as diverse Escherichia coli isolates. With regard to the cytokines, the highest induction levels upon MV exposure were observed for GROα and IL-8, with up to 10- and 15-fold increases in cytokine release compared to mock-treated cells (no MVs). Noteworthy, MVs from E. coli Nissle, a strain that is used as a probiotic and therapeutic agent, also induced a robust cytokine release comparable to those of pathogenic E. coli isolates. In contrast, MVs from several pathogens, such as Klebsiella pneumoniae, V. cholerae, and Yersinia enterocolitica, did not cause any increase in the six cytokines tested.

### IL-8 response evoked by ETEC MVs depends on porins and LPS.

**FIG 2:**
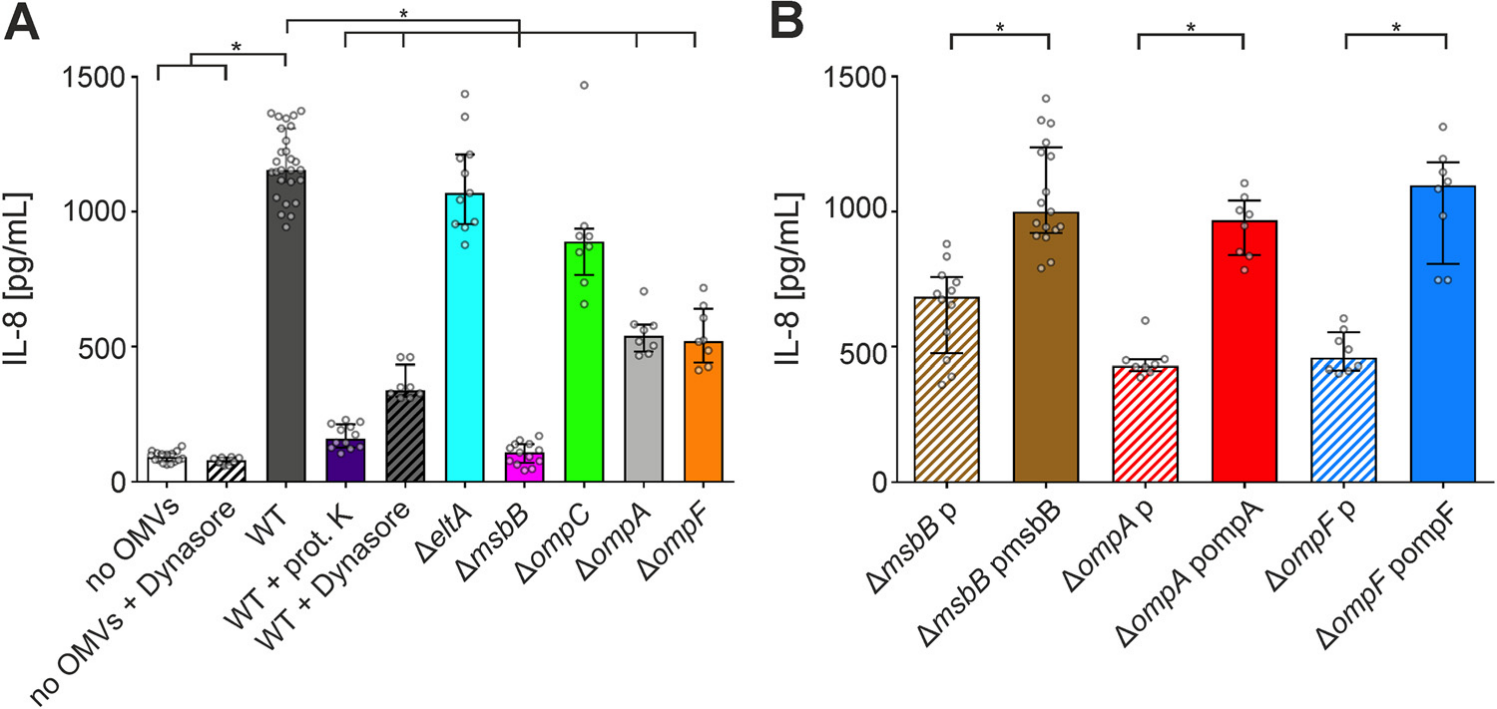
IL-8 response in HT-29 intestinal cells to OMVs derived from ETEC H10407 WT or diverse surface mutants. Cytokine levels were quantified by ELISA in supernatants of HT-29 intestinal cells incubated for 16 h with equal amounts of OMVs. Donor strains of the OMVs are indicated on the *x* axis. (A) HT-29 cells were exposed to OMVs derived from ETEC H10407 (WT) or deletion strains as indicated. In addition, OMVs of the WT were either treated with proteinase K prior to their incubation with HT-29 cells (WT + prot. K) or exposed to HT-29 cells in the presence of the uptake inhibitor dynasore (WT + dynasore). Incubation with saline or the uptake inhibitor dynasore served as negative controls (no OMVs or no OMVs + dynasore). (B) HT-29 cells were exposed to OMVs derived from deletion mutants harboring empty vector (p) or expression plasmids as indicated. (A and B) Data are indicated as the median ± interquartile range (*n* = 16 for no OMVs, *n* = 27 for WT, *n* = 12 for WT + prot. K as well as Δ*msbB* p, *n* = 13 for Δ*msbB*, *n* = 11 for Δ*eltA*, *n* = 17 for Δ*msbB* pmsbB, and *n* = 8 for all other data sets) Asterisks highlight significant differences between respective data sets (*, *P* < 0.05 by Kruskal-Wallis test, followed by Dunn’s *post hoc* test for multiple comparisons or Mann-Whitney U test for single comparisons).

To characterize the inflammatory response elicited by one bacterial MV type in more detail, we chose ETEC H10407 for further analyses as (i) its MVs showed a robust and representative cytokine induction profile among the bacterial MVs with proinflammatory efficacy tested, (ii) its MVs could be isolated with sufficient yield and reproducible quality, and (iii) the strain is genetically engineerable, and several deletion strains are available from our previous work (35). Here, ETEC H10407 is referred to as the wild-type (WT) strain and the corresponding vesicles as OMVs. Among the cytokines, we selected for further analyses IL-8, which showed generally a high induction in HT-29 cells upon MV exposure. MVs are multifactorial complexes with diverse proinflammatory compounds. To narrow the chemical nature of the proinflammatory effector, we analyzed the IL-8 response evoked from HT-29 cells exposed to WT OMVs with and without proteinase K treatment. Interestingly, proteinase K treatment massively reduced the IL-8 release, suggesting that a proteinaceous factor was responsible for the proinflammatory potency of WT OMVs (Fig. 2A). Furthermore, we tested OMVs from a variety of strains with deletion of *eltA*, encoding the catalytic A subunit of LT, or *ompA*, *ompC*, and *ompF*, which encode abundant outer membrane porins. LT is associated with OMVs and can activate the NF-κB signaling pathway (12, 13). However, LT can be excluded here as a relevant proinflammatory factor because MVs from the Δ*eltA* mutant induced a comparable IL-8 response to WT OMVs (Fig. 2A). Among the outer membrane porin deletion strains, OMVs derived from Δ*ompA* and Δ*ompF* strains showed a significantly reduced IL-8 activation compared to WT OMVs. Cell viability was not affected by any of the MVs used, ruling out different IL-8 responses due to cytotoxicity (Fig. S3).

In *trans* expression of OmpA and OmpF in the respective deletion strains restored the proinflammatory potency of the MVs compared to MVs derived from Δ*ompA* and Δ*ompF* strains carrying an empty vector (Fig. 2B). Interestingly, deletion of *msbB*, coding for a secondary lipid A acyltransferase required for synthesis of hexa-acylated LPS (35, 36), led to almost complete inhibition of the IL-8 response (Fig. 2A). In *trans* complementation of MsbB in the Δ*msbB* strain significantly increased the proinflammatory potency of the OMVs compared to OMVs derived from the Δ*msbB* mutant carrying an empty vector (Fig. 2B). OMVs derived from the Δ*msbB* strain have been demonstrated previously to induce a significantly weaker inflammatory response in RAW macrophages than WT OMVs (35). So far, this observation has been attributed to a reduced stimulation of TLR4 via the underacylated, detoxified LPS present in the Δ*msbB* strain (36). In summary, the characterization of MVs derived from defined surface mutants reveal a strong impact by LPS, while the proteinase K digest of WT OMVs suggests a proteinaceous factor promoting the proinflammatory response.

### Uptake of ETEC OMV depends on porins and is blocked by nystatin or dynasore.

Several reports highlight the uptake of bacterial MVs by nonprofessional phagocytic host cells (37–39). Thus, we hypothesized that the observed proinflammatory response evoked by MVs might depend on intracellular signaling and thus internalization of ETEC OMV by host cells rather than depending on recognition via pattern recognition receptors on the host cell surface. Indeed, a recent study identified outer membrane porins OmpU and OmpT from V. cholerae as the essential OMV-associated effectors for cellular uptake in intestinal cells (14).

**FIG 3:**
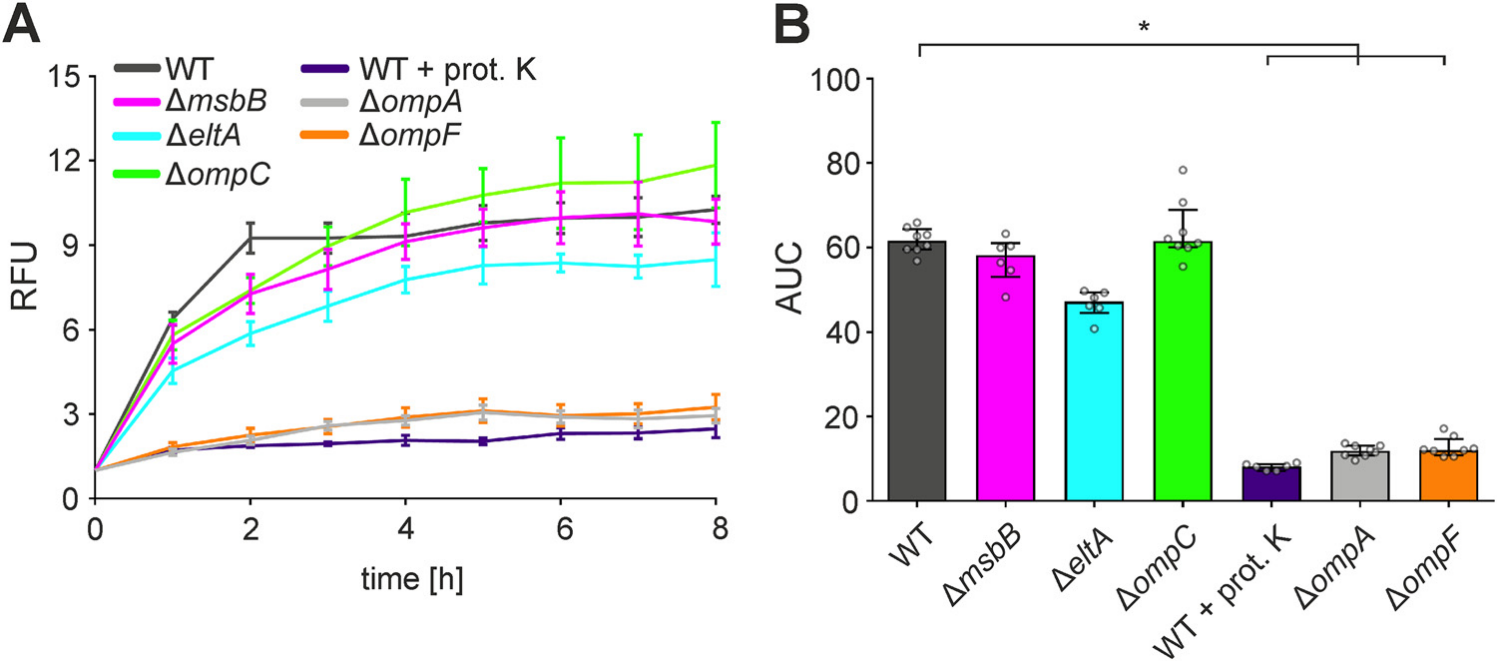
Uptake of OMVs derived from ETEC H10407 WT by HT-29 intestinal cells depends on surface porins OmpA and OmpF. (A) HT-29 intestinal epithelial cells were incubated for 8 h with rhodamine-labeled OMVs derived from the ETEC H10407 WT, Δ*msbB*, Δ*eltA*, Δ*ompC*, Δ*ompA*, or Δ*ompF* strain. Alternatively, OMVs derived from the ETEC H10407 WT were treated with proteinase K prior to their incubation with HT-29 cells (WT + prot. K). Uptake was detected by an increase in relative fluorescence units (RFU) measured every hour. Wells containing rhodamine-labeled OMVs without cells served as a blank. Shown are the mean ± standard deviation, where *n* = 8 for the WT, Δ*ompC*, Δ*ompA*, and Δ*ompF* strains and *n* = 6 for all other data sets. (B) Shown are the median area under the curve (AUC) values ± interquartile range retrieved from the uptake analyses in HT-29. Asterisks highlight significant differences between respective data sets (*, *P* < 0.05 by Kruskal-Wallis test, followed by Dunn’s *post hoc* test).

Along that line, we investigated the uptake of octadecyl rhodamine B chloride (R18)-labeled OMVs derived from ETEC WT and deletion strains into the human intestinal epithelial cell line HT-29, as previously established for vesicles derived from other Gram-negative bacteria (14, 37, 38). OMVs from the ETEC WT were readily taken up by HT-29 cells, reaching maximum levels within 2 to 4 h (Fig. 3A). Uptake of proteinase K-treated WT OMVs was almost completely abolished, indicating that a proteinaceous factor is responsible for the internalization in intestinal epithelial cells. Among the OMVs derived from diverse mutants tested, only OMVs from the Δ*ompA* and Δ*ompF* outer membrane porin deletion strains showed significantly reduced uptake dynamics. Accordingly, the area under the concentration-time curve (AUC) values of uptake assays were significantly reduced for proteinase K-treated WT OMVs as well as for MVs derived from the Δ*ompA* and Δ*ompF* strains in comparison to WT OMVs (Fig. 3B). Notably, MVs derived from the Δ*msbB* strain showed uptake dynamics comparable to those of WT OMVs, excluding an impact of underacylated LPS on MV uptake. In *trans* complementation of OmpA in the Δ*ompA* strain or OmpF in the Δ*ompF* strain significantly enhanced uptake of the MVs in comparison to MVs derived from the Δ*ompA* or Δ*ompF* strains carrying an empty vector, respectively (Fig. S4). Thus, internalization of ETEC OMVs mainly depends on the porins OmpA and OmpF.

**FIG 4:**
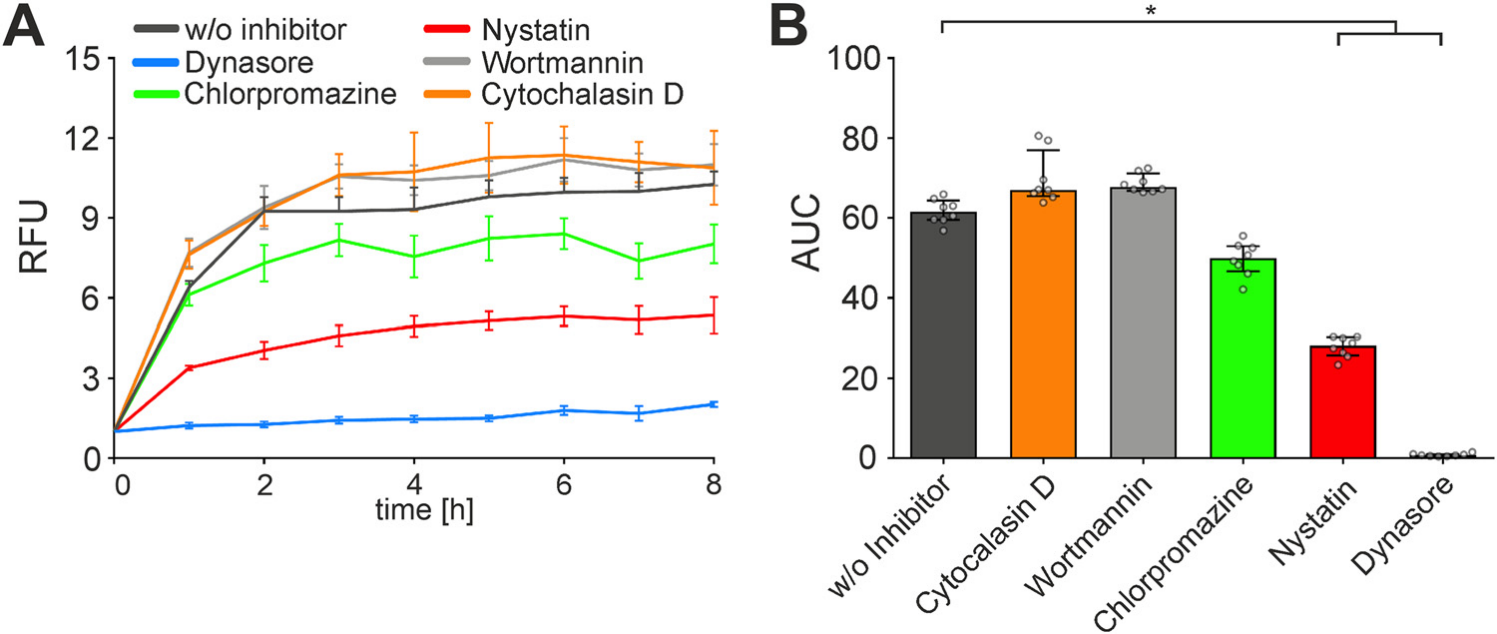
Uptake of OMVs derived from the ETEC H10407 WT by HT-29 intestinal cells is significantly reduced by dynasore or nystatin. (A) HT-29 intestinal epithelial cells were incubated for 8 h with rhodamine-labeled OMVs derived from the ETEC H10407 WT in the presence of uptake inhibitors cytochalasin D, wortmannin, chlorpromazine, nystatin, and dynasore or the solvent DMSO (w/o inhibitor). Uptake is detected by an increase in relative fluorescence units (RFU) measured every hour. Wells containing rhodamine-labeled OMVs from the WT without cells served as a blank. Shown is the mean ± standard deviation, where *n* = 8. (B) Shown are the median area under the curve (AUC) values ± interquartile range retrieved from the uptake analyses in HT-29. Asterisks highlight significant differences between respective data sets (*, *P* < 0.05 by Kruskal-Wallis test, followed by Dunn’s *post hoc* test).

Next, we used a comprehensive set of commercially available uptake inhibitors covering the most relevant entry routes that have been reported so far for bacterial MVs (37, 40–43) to identify the pathways involved in the uptake of ETEC OMVs (Fig. 4). To the best of our knowledge, changes in the OMV composition upon presence of any of these uptake inhibitors have not been reported. Addition of wortmannin (inhibition of macropinosome closure), cytochalasin D (inhibition of membrane fusion), chlorpromazine (inhibition of clathrin-dependent endocytosis), and amiloride (inhibition of micropinocytosis) had no significant effect on the uptake dynamics of ETEC OMVs. In contrast, addition of nystatin, which intercalates and disrupts cholesterol-rich membrane domains affecting caveolin-mediated endocytosis and lipid raft formation, resulted in a significant decrease of OMV uptake compared to the solvent (dimethyl sulfoxide [DMSO])-treated control (Fig. 4). Moreover, dynasore, which inhibits dynamin GTPase activity, preventing clathrin-dependent and caveolin-mediated endocytosis, abolished MV uptake almost completely. The strong inhibitory effect of nystatin or dynasore strongly suggests that ETEC OMVs are predominantly taken up by caveolin-mediated endocytosis, which is affected by both compounds.

Importantly, the presence of dynasore, also significantly reduced the IL-8 response in HT-29 cells upon contact with OMVs derived from ETEC WT (Fig. 2). This indicates that blockage of uptake abolishes the proinflammatory cytokine induction, which consequently likely depends on intracellular recognition of ETEC MVs in intestinal epithelial cells. Moreover, OMVs derived from the Δ*msbB* strain show similar uptake dynamics but strongly reduced IL-8 activation compared to WT OMVs. As mentioned above, the ETEC WT produces hexa-acylated LPS, whereas the Δ*msbB* mutant synthesizes underacylated LPS (35, 36). Surface mutants of K. pneumoniae show compensatory effects, which can modulate the OMV cargo (44, 45). Thus, we quantified amounts of protein and LPS in OMV preparations derived from the ETEC WT and mutants (see Table S1 in the supplemental material). Aside from variations in MV yields highlighted by different amounts of biomass, the protein and LPS ratio can change in surface mutants, as indicated by the different protein/LPS ratios. Overall, OMVs from surface mutants carry more LPS than WT OMVs, which is concordant with earlier reports demonstrating elevated LPS levels in OMVs from *K. pneumonia* upon depletion of porins (44). Thus, the low proinflammatory potency observed for OMVs from the Δ*msbB* mutant cannot be explained by lower LPS levels. These findings already point toward an intracellular recognition of the lipid A moiety of the LPS inducing a proinflammatory response.

### Inflammatory response induced by ETEC OMVs in intestinal epithelial cells is mediated by caspases and RIPK2.

**FIG 5:**
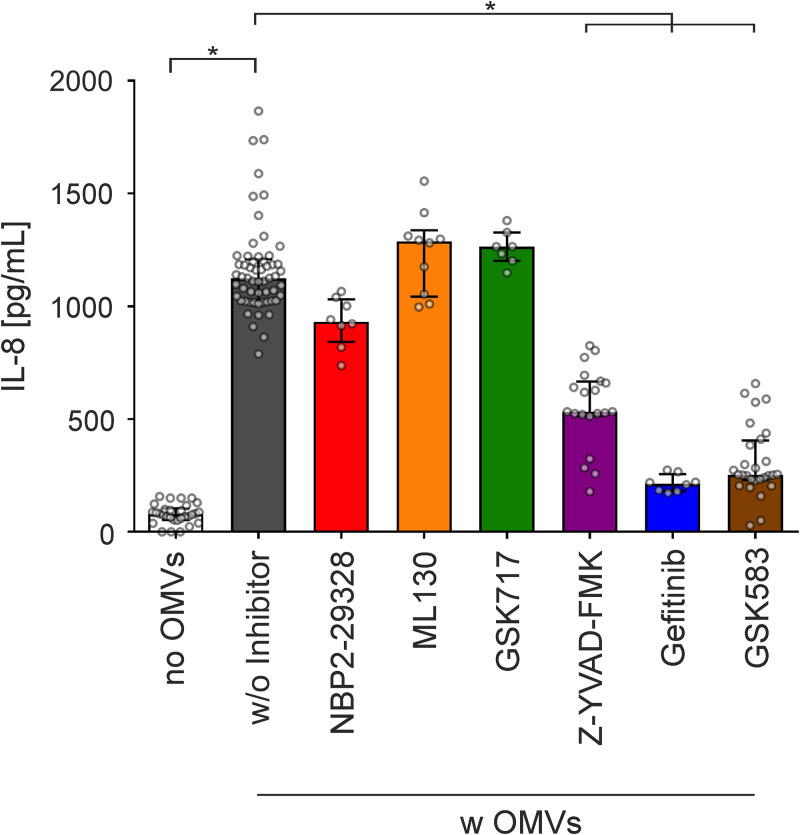
RIPK2 and caspase inhibitor block IL-8 response provoked by OMVs from the ETEC H10407 WT strain in HT-29 intestinal cells. Cytokine levels were quantified by ELISA in supernatants of HT-29 intestinal cells incubated for 16 h with OMVs derived from the ETEC H10407 WT (w OMVs) in the presence of solvent DMSO (w/o inhibitor) or inhibitors for MyD88 (NPB2-29328), Nod1 (ML130), Nod2 (GSK717), caspase (Z-YVAD-FMK), or RIPK2 (gefitinib and GSK583) cascades. Incubation with solvent DMSO served as the negative control (no OMVs). Data are indicated as the median ± interquartile range (*n* = 30 for no OMVs, *n* = 57 for OMVs w/o inhibitor, *n* = 8 for NPB2-29328 and gefitinib, *n* = 10 for ML130, *n* = 7 for GSK717, *n* = 20 for Z-YVAD-FMK, and *n* = 28 for GSK583). Asterisks highlight significant differences between respective data sets (*, *P* < 0.05 by Kruskal-Wallis test, followed by Dunn’s *post hoc* test).

To decipher the relevant host cell pathways involved in the observed proinflammatory cytokine induction, we used a diverse set of inhibitors along with WT OMVs exposed to HT-29 cells (Fig. 5). The major LPS sensing pathway relies on TLR4 (46, 47). However, a pivotal role of TLRs in sensing OMV-associated MAMP can be excluded, as an inhibitor of MyD88 (NPB2-29328), representing the key adaptor protein required by most TLRs to activate NF-κB and proinflammatory cytokine release, did not abrogate the secretion of IL-8 (Fig. 5). Based on the internalization dependency of the IL-8 release, we sought to determine the role of cytosolic NOD1 and NOD2 receptors, which are constitutively expressed in HT-29 cells (48) or the cytosolic LPS sensing pathway, which depends on human caspase-4/5 or caspase-11 in mice (49). Neither inhibition of NOD1 (by ML130) nor that of NOD2 (by GSK717) resulted in a substantial decrease of the WT OMV-mediated IL-8 release (Fig. 5). In contrast, treatment of HT-29 cells with Z-YVAD-FMK, a cell-permeable, irreversible inhibitor of human caspase-1, -4, and -5, significantly decreased WT OMV-mediated IL-8 secretion in intestinal epithelial cells (Fig. 5). Furthermore, inhibition of RIPK2 with the tyrosine kinase inhibitor gefitinib (50) or the RIPK2-specific inhibitor GSK583 (51) significantly reduced the MV-mediated IL-8 secretion (Fig. 5). Intracellular sensing of LPS by caspase-4/5 can result in noncanonical inflammasome activation, which requires binding of the lipid A moiety of the LPS to CARD of the procaspases, promoting their autoproteolytic cleavage, maturation, and oligomerization (49). Furthermore, recent studies have demonstrated that stimulation of HEK293T cells with crude LPS can also drive NF-κB activation and subsequent proinflammatory cytokine release via caspase-1 in an RIPK2-dependent manner (52–55). This noncanonical signaling does not require proteolytic activity of caspase-1, but rather relies on protein-protein interactions of procaspase-1 with RIPK2 via their CARD (52). A direct target for caspase-1 and caspase-4/5 is the processing and secretion of IL-18 (49, 53). Indeed, HT-29 intestinal cells exposed to OMV derived from ETEC WT showed significantly elevated IL-18 secretion compared to mock-treated control cells (Fig. S5). Concordant with the IL-8 response, HT-29 cells exposed to OMVs derived from the Δ*msbB* mutant showed no detectable IL-18 induction, while in *trans* expression of MsbB restored the proinflammatory potency of the OMVs compared to OMVs derived from Δ*msbB* carrying an empty vector (Fig. S5). Thus, full acylation of the lipid A moiety of OMV-associated LPS is essential for induction of a proinflammatory response (i.e., release of IL-8 and IL-18).

**FIG 6:**
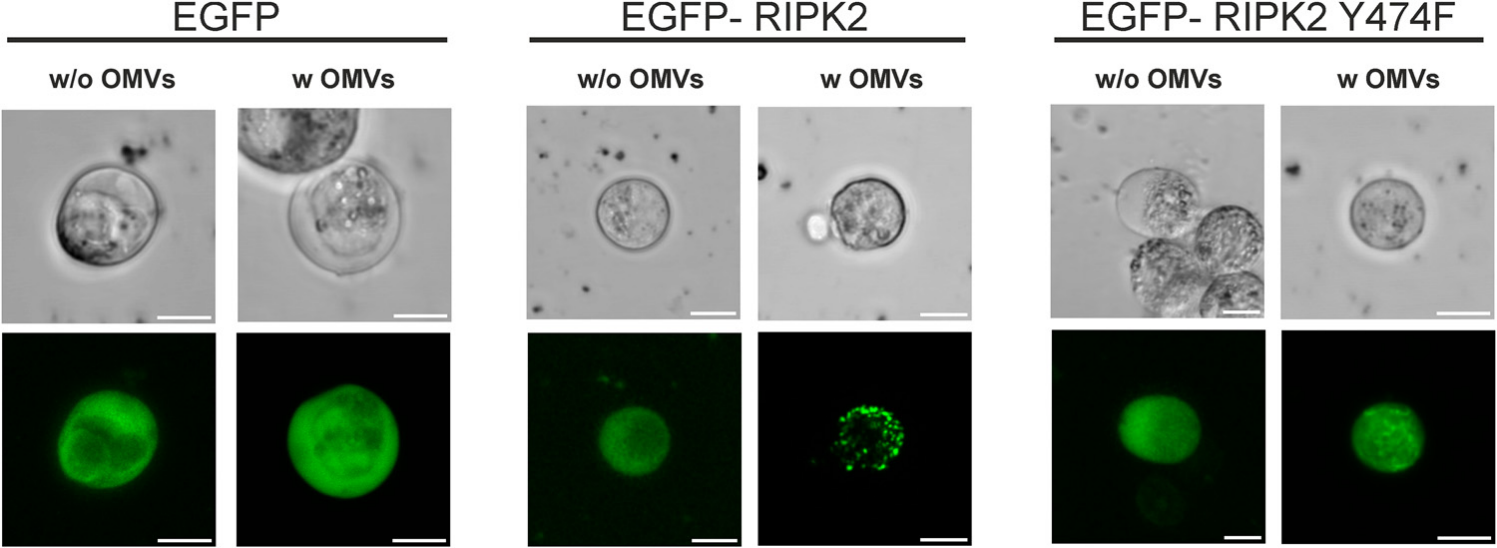
Visualization of RIPosome formation in HT-29 intestinal cells upon exposure to OMVs from the ETEC H10407 WT strain. Shown are transmission (top row) and fluorescence (bottom row) micrographs of HT-29 cells transfected with expression plasmids for EGFP (control), EGFP-RIPK2, or EGFP-RIPK2 Y474F after 16 h of incubation with OMVs from the ETEC H10407 WT (w OMVs) or saline (w/o OMVs). Dead cells were visualized by propidium iodine staining and excluded from RIPosome formation analyses. (A representative example is provided in Fig. S6.) Scale bar = 10 μm.

**FIG 7:**
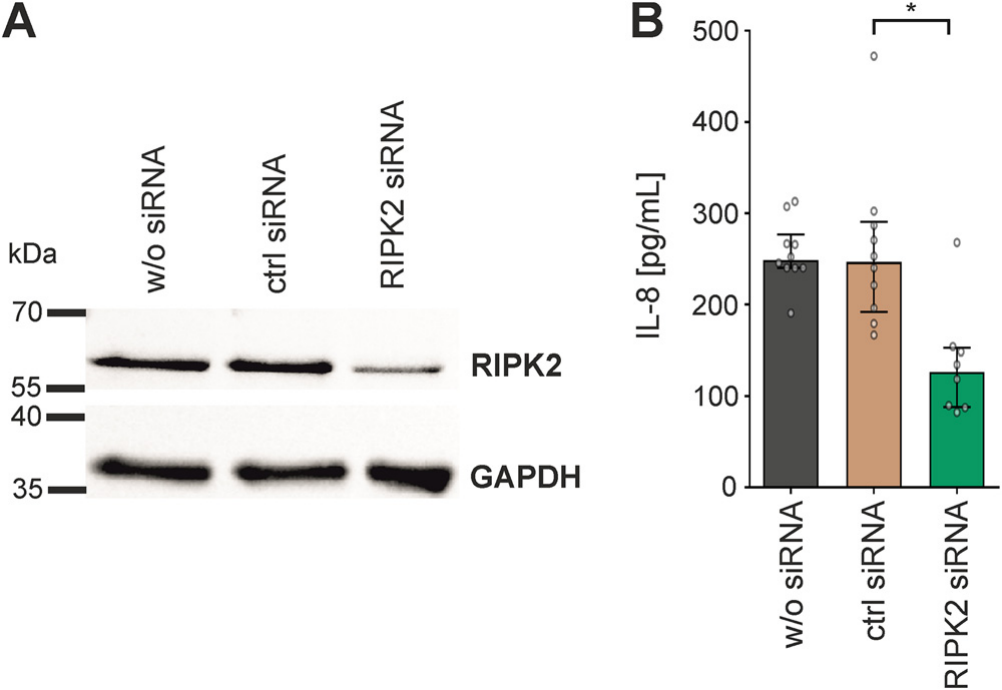
Knockdown of RIPK2 reduces IL-8 release induced by OMVs from the ETEC H10407 WT strain in HT-29 intestinal cells. (A and B) HT-29 cells were treated for 48 h with RIPK2 siRNA or a nontargeting control (ctrl) siRNA or left untreated (w/o siRNA), followed by a 16-h exposure to OMVs derived from the ETEC H10407 WT. (A) Immunoblot analysis of whole-cell lysates from HT-29 cells treated as described above. Immunoblots probed with anti-RIPK2 antibody and GAPDH-antibody as loading control are shown. (B) IL-8 release was quantified by ELISA in supernatants of HT-29 cells treated as described above. Data are indicated as the median ± interquartile range (*n* = 10 for w/o siRNA and ctrl RNA as well as *n* = 8 for RIPK2 siRNA). Asterisks highlight significant differences between respective data sets (*, *P* < 0.05 by Kruskal-Wallis test, followed by Dunn’s *post hoc* test).

Intracellular signaling of OMV-associated LPS liberated from internalized OMVs via the murine caspase-11 pathway in bone marrow-derived macrophages and HeLa cells was recently suggested by Vanaja et al. (56), while evidence for an RIPK2-dependent signaling of LPS delivered via OMVs was hitherto lacking. Thus, we characterized the RIPK2 activation upon exposure with WT OMVs in more detail. In HeLa cells, RIPK2 forms high-molecular-weight complexes, also known as RIPososmes, upon infection with invasive bacterial pathogens: i.e., Shigella flexneri and enteropathogenic E. coli (EPEC) (57, 58). This RIPosome formation resulting from RIPK2 activation depends on autophosphorylation of RIPK2 at position Y474 (57). To investigate RIPK2 activation in intestinal cells upon exposure with WT OMVs, we transfected HT-29 cells with expression plasmids for enhanced green fluorescent protein (EGFP)-RIPK2 and EGFP-RIPK2 Y474F as well as EGFP as control (59, 60). EGFP-RIPK2 showed uniform cytoplasmic distribution in mock-treated cells, which changed to dot-like structures indicating formation of cytosolic RIPososme complexes upon exposure to MVs (Fig. 6). In neither the absence nor presence of MVs was such complex formation observed in intestinal cells expressing the EGFP-RIPK2 Y474F phosphorylation mutant or the EGFP control. Moreover, silencing of RIPK2 in HT-29 cells by siRNA transfection resulted in significant decrease of IL-8 upon exposure to WT OMVs in comparison to cells transfected with control siRNA (Fig. 7). These results suggest that RIPK2 is a key factor in the proinflammatory response induced in HT-29 cells upon exposure to MVs. To summarize, our experiments strongly suggest that the internalization of WT OMVs activates caspase- and RIPK2-dependent pathways to promote proinflammatory responses.

## DISCUSSION

It is becoming increasingly evident that bacterial MVs can promote diverse immunomodulatory responses in host cells. These are complex as given by the different compositions of bacterial vesicles, which depend on the donor bacterium and mechanism of vesicle release: e.g., blebbing or explosive cell lysis (1). Furthermore, recognition of MVs by host cells via PRRs might differ between tissues and cell type. Currently, the number of studies investigating host cell responses upon exposure to bacteria MVs is limited, and comparability is hampered by the use of different bacterial cultivation conditions, MV isolation protocols, and host cell types. This comparative study provides a first comprehensive proinflammatory cytokine profiling of the intestinal epithelial cell line HT-29 upon exposure to MVs derived from 32 intestinal bacterial strains. The induced cytokine profile of intestinal epithelial cells exposed to different MVs varied in quantity and quality, depending on the MV used. This suggests that each MV species can induce specific immunomodulatory activity in host cells—almost like a unique fingerprint. It is likely that changes in gut microbiome composition and diversity affect the MV population released by gut bacteria. Consequently, alterations in the gut microbiota can change the proinflammatory potential of the MV population in the gut, which in turn shapes inflammatory responses. Thus, it is tempting to speculate that alterations of the MV composition in the gut can initiate or exacerbate the outcome of inflammatory intestinal diseases, which needs to be addressed in future studies.

Notably, OMVs from the probiotic strain E. coli Nissle demonstrated relatively high proinflammatory potency in our cell culture assays. Although suggested as alternative therapy to maintain remission in ulcerative colitis patients with comparable efficacy to the established gold standard therapy based on mesalazine, a recent study indicates adverse effects in 15% of the patients receiving E. coli Nissle within the first few weeks (61, 62). Concordantly, severe adverse effects by E. coli Nissle were reported in a mouse model with disturbed microbiota and impaired adaptive immunity (63).

Further research needs to address the individual immunomodulatory potential of bacterial MVs and characterize the underlying molecular mechanisms to highlight common principles as well as species-specific differences. Here, we focused on the proinflammatory activity of ETEC OMVs, demonstrating robust cytokine induction in intestinal epithelial cells. Moreover, ETEC 10407 can be genetically modified, can be easily cultivated under laboratory conditions, and demonstrates a robust OMV production (35). Our findings unravel a two-step recognition of ETEC OMVs by intestinal epithelial cells, requiring a porin-dependent internalization, followed by intracellular recognition of LPS. In detail, inhibitor analyses identified caveolin-mediated endocytosis to be the preferred uptake pathway, which is facilitated by presence of the two outer membrane porins OmpA and OmpF.

Similar to those of many Gram-negative species, OmpA is a major porin in the outer membrane of E. coli (13, 64). Notably, OMV-associated OmpA can contribute to interactions of Gram-negative MVs with host cells. For example, OmpA associated with E. coli OMVs facilitates invasion by interactions with the surface receptor Ecgp on brain microvascular endothelial cells (65, 66). OmpA located on Acinetobacter baumannii MVs causes mitochondrial fragmentation and cytotoxicity (67). In E. coli, expression of OmpF is positively regulated by the EnvZ/OmpR two-component system, which is likely to be activated during *in vivo* colonization via the envelope stress triggered by host-derived factors such as bile salts (68, 69). Thus, the presence of these two porins on OMVs released from ETEC during intestinal colonization is likely, although the MVs used in this study were isolated from *in vitro* cultures grown in brain heart infusion (BHI) broth.

In the case of K. pneumoniae, loss of porins is associated with compensatory effects, such as enhanced transcription of other porins and increased LPS content (44, 45). Thus, we cannot exclude that OMV internalization into host cells requires other effectors, which are altered upon loss of OmpA or OmpF. Nonetheless, the similarity in uptake dynamics of OMVs from the Δ*ompC* and ETEC WT strains argues against a general internalization defect achievable by mutation of any porin, but rather points toward a more specific mechanism depending on presence of OmpA and OmpF. Even though not documented in literature, we cannot exclude that loss of porins in ETEC changes the acylation state of LPS in the OMVs. However, proteinase K treatment of ETEC WT OMVs, which is unlikely to affect LPS chemistry, abrogated internalization, strongly suggesting that host cell uptake depends on proteinaceous factors.

Based on their multiple immunostimulatory compounds, bacterial MVs have been shown to interact with a variety of surface-exposed and cytoplasmic PRRs to promote inflammatory responses (23, 70). For example, lipoproteins in MVs released by mycobacterial species are recognized by TLR2, flagellins present on OMVs from EHEC O157 can activate TLR5, and nucleic acids associated with MVs from S. aureus or Porphyromonas gingivalis can activate TLR7, -8, and -9 (71–74). Recognition of MV-associated effectors by TLRs can also be beneficial, as shown for polysaccharide A-containing OMVs from the commensal Bacteroides fragilis, which are sensed by dendritic cells via TLR2 and promote anti-inflammatory responses (27). Particularly, LPS associated with OMVs from Gram-negative bacteria can activate the TLR4 on the surface of host cells as well as intracellular TLR4 (75–77). Importantly, the adaptor protein Myd88 is pivotal for downstream signaling of bacterial effectors via TLRs to ultimately activate NF-κB (78). A dominant role in sensing ETEC OMVs via TLRs can be excluded as the presence of an Myd88 inhibitor had no effect on the proinflammatory response in intestinal epithelial cells upon exposure of ETEC OMVs. Moreover, bacterial MVs derived from diverse pathogens were shown to activate cytoplasmic NOD1 or NOD2 in various host cells, such as epithelial cells and monocytes. For example, peptidoglycan present in OMVs derived from Helicobacter pylori, Neisseria gonorrhoeae, and Pseudomonas aeruginosa is detected by NOD1, resulting in proinflammatory responses (23). Activation of NOD1 and NOD2 was reported for MVs from the intestinal pathogen V. cholerae as well as periodontal pathogens Aggregatibacter actinomycetemcomitans and P. gingivalis (74, 79, 80). However, in this work inhibition of neither NOD1 nor NOD2 reduced the ETEC OMV-dependent proinflammatory cytokine release in intestinal epithelial cells, which excludes a dominant role of these intracellular receptors in the recognition of ETEC OMVs.

Our results rather indicate that LPS is the dominant proinflammatory bacterial effector and that it is recognized by caspase- and RIPK2-dependent cytosolic pathways upon internalization of the ETEC OMVs in intestinal epithelial cells. MVs derived from the Δ*msbB* mutant harboring underacylated LPS showed comparable uptake dynamics, but significantly reduced proinflammatory cytokine induction. Modifications of LPS in K. pneumoniae and P. gingivalis have been reported to affect the protein cargo of OMVs, which may change the inflammatory potential of OMVs (45, 81, 82). It should be noted that these LPS modifications depend on changes or loss of the O antigen, which alters the net charge of the LPS and likely affects protein deposition in OMVs by changed electrostatic interactions. In contrast, this study used a Δ*msbB* mutant with underacylated lipid A. Earlier reports focusing on OMVs as vaccine candidates comprehensively characterized the OMV protein and LPS profile of the ETEC WT and a Δ*eltA* Δ*msbB* double mutant (35). The only notable difference was a missing protein band in OMVs derived from the Δ*eltA* Δ*msbB* mutant at the height of EltA. Thus, major changes in the protein cargo of OMVs derived from Δ*msbB* can be excluded. The similar protein composition of OMVs is also in line with comparable internalization dynamics observed for OMVs from the ETEC WT and Δ*msbB* strains, reinforcing the involvement of proteinaceous factors for host cell uptake.

In summary, hexacylated lipid A moieties seem to be dispensable for uptake, but are readily recognized by intracellular receptors promoting proinflammatory responses. Recent reports highlight that LPS can bind to several caspases (83, 84). Murine procaspase-11 and human procaspase-4/5 recognize LPS via binding of the lipid A moiety to CARD motifs (49), while LPS detection via caspase-3/7 depends on the O-antigen region (85). In the case of ETEC OMVs, the binding of the lipid A moiety of MV-delivered LPS to CARD motifs from caspases seems to be a relevant factor, as full acylation of the LPS is required for a proinflammatory response, as seen by the IL-8 and IL-18 secretion. On the one hand, this can lead to procaspase-4/5 activation, followed by proteolytic cleavage, maturation, and oligomerization of the caspases leading to noncanonical inflammasome activation (56). On the other hand, our data suggest that hexa-acylated lipid A provided by LPS from OMVs can also activate the previously reported interaction of procaspase-1 with RIPK2, which results in activation of NF-κB (52). Based on the inhibitor analyses and knockdown assays, the RIPK2-dependent pathway seems to be pivotal for proinflammatory cytokine induction in intestinal epithelial cells (i.e., HT-29). This is so far surprising as, to the best of our knowledge, in epithelial cells a clear role for RIPK2 in intercellular LPS sensing has not been reported. The fact that we observed formation of RIPosomes, which we so far only associated with NOD1/2-dependent NF-κB and autophagy induction, is indicative that the WT OMV induced a novel signaling pathway. This was blocked by the RIPK2-specific inhibitor GSK583, which affects RIPK2 binding to the X-linked inhibitor of apoptosis protein (XIAP) (86), indicating that the pathway depends on the classical RIPK2 activation of IκB kinase (IKK), which is involved XIAP-mediated ubiquitination (87). As the caspase inhibitor we used (Z-YVAD-FMK) is not specific for caspase-4 but also targets caspase-1, further work needs to identify the caspases involved in the pathway. Caspase-1-mediated NF-κB activation by RIPK2 is counteracted by the presence of the inflammasome adaptor protein ASC (55), suggesting that this pathway is particular active in cells with low ASC expression, such as the intestinal epithelial cells used herein.

In combination with previous reports highlighted above, our results indicate that different bacterial MVs can be sensed by diverse host receptors and activate distinct signaling cascades. Here, we demonstrate that proinflammatory response of intestinal epithelial cells upon exposure to ETEC OMVs requires a porin-dependent internalization, followed by a novel caspase- and RIPK2-dependent cytosolic recognition of the bacterial LPS. More studies focusing on host responses to diverse MV species will be necessary to identify unique immunomodulatory features as well as common principles of the bacterial MV-host interaction.

## MATERIALS AND METHODS

### Bacterial strains and growth conditions.

The bacterial strains and plasmids used in this study are listed in Table S2 in the supplemental material, and oligonucleotides are listed in Table S3. From Fig. 2 onwards, a spontaneous streptomycin-resistant (Sm^r^) mutant of the enterotoxigenic Escherichia coli (ETEC) clinical isolate H10407 served as the wild-type (WT) strain. E. coli strains DH5α λpir and SM10 λpir were used for genetic manipulations (88). Unless stated otherwise, bacteria were grown at 37°C in lysogeny broth (LB) or on LB agar for genetic manipulations or in brain heart infusion (BHI) broth or on BHI agar in the case of membrane vesicle isolation. Antibiotics and other supplements were used in the following final concentrations: streptomycin (Sm), 100 μg/mL; ampicillin (Ap), 50 μg mL in combination with other antibiotics, otherwise 100 μg/mL; kanamycin (Km), 50 μg mL; sucrose, 10%; and arabinose, 0.2%.

### Construction of in-frame deletion mutants and expression plasmids.

The isolation of chromosomal DNA, PCRs, the purification of plasmids or PCR products, and the construction of suicide and expression plasmids as well as the subsequent generation of deletion mutants were carried out as described previously (89, 90). Qiagen plasmid kits were used for isolation of plasmid DNA, and Qiaquick Gel extraction and Qiaquick PCR purification kits (Qiagen) were used for purification of DNA fragments. PCRs for subcloning were carried out using the Q5 high-fidelity DNA polymerase (NEB), while *Taq* DNA polymerase (NEB) was used for all other PCRs. Strains used for genetic manipulation were cultivated with aeration in lysogeny broth (LB).

Constructions of in-frame deletion mutant was carried out as described by Donnenberg and Kaper (91). Briefly, ~800-bp PCR fragments located upstream and downstream of the respective gene were amplified using the oligonucleotide pairs X_Y_1 and X_Y_2 as well as X_Y_3 and X_Y_4 (Table S3), where X represents the gene and Y the respective digestion enzyme. After digestion of the PCR fragments with the appropriate restriction enzyme (NEB) indicated by the name of the oligonucleotide, they were ligated into pCVD442, which was digested with the appropriate restriction enzymes. Unless noted otherwise, ligation products were transformed into DH5α λpir, and Ap^r^ colonies were characterized for the correct constructs by PCR (and restriction analysis). The obtained corresponding knockout plasmids are listed in Table S1.

To obtain deletion strains, generated derivatives of pCVD442 were transformed into E. coli Sm10 λpir and conjugated into ETEC H10407. Exconjugants were purified by Sm^r^/Ap^r^ selection. Sucrose selection was used to obtain Ap^s^ colonies, and chromosomal deletions/replacements were confirmed by PCR.

For construction of the plasmids expressing *msbB* as well as *ompA* and *ompF*, the genes with a C-terminal His tag were amplified with PCR using the oligonucleotide pairs X_Y_fw and X_HIS_Y_rev, where X represents the gene, HIS the C-terminal His tag, and Y the restriction site (Table S3). After digestion with appropriate restriction enzymes, fragments were ligated into a similarly digested pBAD18-Kan vector and transformed into E. coli DH5α λpir. The plasmids were isolated and brought into the respective ETEC H10407 strains by transformation. Clones were verified by colony PCR.

### Isolation of membrane vesicles from intestinal bacterial strains.

OMVs were essentially isolated as described previously, with minor modifications (92). Briefly, overnight cultures of the respective strains were grown with aeration (180 rpm on an Infor shaker) or anaerobically (GasPak EZ systems; BD) in BHI broth to ensure sufficient growth for the diverse bacterial species analyzed (see Table S1 for details). The respective cultures were diluted (1:100) in BHI medium and grown at 37°C either with aeration for 8 h or overnight anaerobically (GasPak EZ systems; BD). The cells were then removed from the supernatant by centrifugation (9,000 × *g*, 15 min) and subsequent sterile filtration (0.22-μm pore). The OMVs present in the supernatant were pelleted through subsequent ultracentrifugation (150,000 × *g*, 4°C, 4 h), resuspended in appropriate volumes of saline to generate an OMV suspension, and further purified by density gradient ultracentrifugation as previously published (93). Briefly, MVs were applied onto an isopycnic OptiPrep-iodixanol (Sigma-Aldrich) density gradient (1.4 mL each of 55%, 50%, 45%, 40%, 35%, 30%, and 25% iodixanol in double-distilled water [ddH_2_O]) and ultracentrifuged (150,000 × *g*, 17 h, 4°C, SW41 Ti rotor). Gradient fractions (1 mL) were collected from the top and diluted to 35 mL in 10 mM Tris/HCl (pH 7.4). Each diluted gradient fraction was pelleted by ultracentrifugation (144,000 × *g*, 4 h, 4°C, SW 32 Ti rotor) and resuspended in saline. Fractions containing high MV concentrations were confirmed by SDS-PAGE (data not shown) and pooled. Samples were stored at −20°C until further use.

### Protein quantification.

Protein concentrations of purified MVs were finally determined by Bradford assay with Bio-Rad Laboratories protein assay dye reagent according to the manufacturer’s manual. It should be noted that the protein concentrations determined in this study refer to quantifications without MV lysis. Thus, the determined amounts cannot be seen as total MV protein amounts as internal proteins might have been not accurately measured. Reanalyses of MV samples from diverse bacteria used in this study (i.e., B. fragilis, E. cloacae, ETEC H1047, E. coli Nissle, K. oxytoca, *S.* Typhimurium, V. cholerae, Bifidobacterium bifidum, L. acidophilus, and E. faecalis), with and without SDS (0.1% for 10 min), indicate that higher protein concentrations upon SDS lysis of MVs can be measured (Table S4). The average fold increases upon SDS exposure were quite similar for all MV types analyzed and ranged between 1.28 (for E. cloacae) and 1.45 (for *S.* Typhimurium) in comparison to protein quantifications without SDS. Thus, although the exact total protein amount might not have been determined, the error appears to be of a similar magnitude for all MV samples tested and therefore does not have a massive impact on interpretation of different proinflammatory potencies observed for the various MVs tested. Furthermore, proteins outside the MVs are more likely to interact with host cells. Therefore, protein measurements without lysis might be a more reliable method to determine the amounts of bioactive proteins relevant for host cell interactions.

### LPS quantification.

To quantify the lipopolysaccharide content of OMVs, Purpald assays were performed as described previously, using 3-deoxy-d-mannooctulosonic acid (Kdo) (Sigma-Aldrich) as a standard (7).

### Rhodamine staining of OMVs.

One-milligram protein equivalents of isolated OMVs were diluted in staining buffer (50 mM Na_2_CO_3_, 100 mM NaCl [pH 9.2]) to a final volume of 1 mL. After addition of 100 μL octadecyl rhodamine B chloride (R18) (Thermo Fisher) to a final concentration of 0.5 mg/mL, OMVs were stained in the dark with constant agitation over night at room temperature. Finally, R18-labeled OMVs were subjected to density gradient purification as described previously (7). Density gradient-purified R18-labeled OMVs were quantified by Bradford assay for the amount of protein to allow use of equal amounts in the uptake assays.

### Cell culture conditions.

HT-29, HT-29 MTX, or Caco-2 cells (intestinal epithelial cells) were grown in T-175 tissue culture flask, containing Dulbecco's modified Eagle's medium plus nutrient F-12 (DMEM–F-12) medium (Gibco, USA) supplemented with 10% fetal bovine serum, penicillin G, and glutamate at 37°C in a CO_2_ incubator. In the case of internalization studies, intestinal epithelial cells were seeded at a concentration of ~1 × 10^5^ cells/well 24 h prior to the uptake assay in a black 96-well plate (see “Uptake assay”).

For cytokine assays, intestinal epithelial cells were seeded in 24-well tissue culture plates at a concentration of 6 × 10^5^ cells/well and cultivated for 24 h in DMEM–F-12 medium supplemented with glutamate and penicillin G, but without fetal bovine serum during the treatment. Then, intestinal epithelial cells were washed once with phosphate-buffered saline (PBS), and the medium was replaced with MVs (from 10 ng/mL to 10 μg/mL protein equivalents in case of Fig. S2, otherwise always 100 ng/mL protein equivalent) resuspended in DMEM–F-12. In case of proteinase K treatment, MVs were incubated overnight at 55°C with 100 μg/mL proteinase K, followed by inactivation for 10 min at 65°C prior to the addition in cell culture.

For inhibition assays, HT-29 cells (6 × 10^5^ cells/well) were treated for 3 h with or without inhibitor for NOD1 (ML130; 10 μM) (Sigma-Aldrich), NOD2 (GSK717; 10 μM) (Sigma-Aldrich), MyD88 (NBP2-29328; 10 μM) (Novus Biological), tyrosine kinases (gefitinib; 10 μM), caspase-1,4,5 (Z-YVAD-FMK; 10 μM) (Sigma-Aldrich), and Rip2 kinase (GSK583; 5 μM) (Sigma-Aldrich) in DMEM–F-12 medium supplemented with glutamate and penicillin G, but without fetal bovine serum, before MVs (100 ng/mL protein equivalent) were added. Addition of DMSO served as a solvent control for the inhibition assays. After incubation for 16 h with MVs, the supernatant was removed and stored at −20°C for Luminex analysis or enzyme-linked immunosorbent assay (ELISA), which were performed according to the manufacturer’s protocol.

### Cell viability assays.

3-(4,5-Dimethyl-2-thiazolyl)-2,5-diphenyl-2H-tetrazolium bromide (MTT) cell viability assays were routinely performed at the end of the cell culture assays (94, 95). Briefly, MTT (5 mg/mL) (Invitrogen) was diluted 10-fold in DMEM–F-12 medium without fetal bovine serum (FBS). One hundred microliters of this solution was added to each well, and the well was incubated for 3 h at 37°C under 5% CO_2_. After incubation, supernatants were carefully removed, and 50 μL of DMSO was then added to each well to solubilize the formazan product. The absorbance was measured at 590 nm using a plate reader. Cell viability was calculated as the ratio of the treated cells to cells with solvent control (i.e., no MVs or OMVs).

### Luminex analysis.

Supernatants of HT-29, HT-29 MTX, and Caco-2 cells after exposure to MVs were analyzed by Luminex analyses for the amounts of 23 different cytokines reported to be expressed in intestinal epithelial cells and related to chronic intestinal diseases like inflammatory bowel disease (IBD) (CCL2/MCP-1, CCL5/RANTES, CCL7/MCP-3, CCL11/eotaxin, CCL20/MIP3a, CCL22/MDC, CCL28/MEC, CXCL1/GROα, CXCL2/MIP2, CXCL5/ENA-78, CXCL9/MIG, CXCL10/IP-10, CXCL11/ITAC-1, CX3CL1/fractaline, tumor necrosis factor alpha [TNF-α], granulocyte-macrophage colony-stimulating factor [GM-CSF], IL-6, IL-8, IL-18, IL-25 [IL-17E], TGF1-b1, gamma interferon [IFN-γ], and TSLP). Supernatants of cells mock treated by addition of an equivalent volume of saline served as negative control (no MVs). Luminex analyses were performed at the Core Facility Imaging of the Medical University of Graz, Graz, Austria (coordinator Heimo Strohmaier).

### Cytokine ELISA.

ELISA was performed to measure the inflammatory molecules in the cell culture supernatants. ELISAs for quantification of CXCL5, CXCL8, and CXCL10 were conducted using capture and secondary antibodies from Biolegend, respectively (526304, 526202, 514704, 514602, 524402, and 519403). Recombinant human CXCL5, CXCL8, and CXCL10 from Biolegend served as standards (573409, 570909, and 573509). ELISAs for quantification of CCL20, CXCL1, and CCL20 were performed using the CCL20/Mip3-alpha ELISA MAX Deluxe Set kit from Biolegend (441406), the CXCL1/Gro-alpha DuoSet ELISA kit (DY275), or the CCL22/MDC DuoSet ELISA kits (DY336) from Bio-Techne. ELISAs were essentially performed according to the manufacturers’ protocols. Briefly, flat-bottom ELISA plates were coated overnight with the designated capture antibody diluted in carbonate coating buffer (100 μL) at 4°C. In each washing step, the plate was washed 3 times with PBS containing 0.05% Tween (PBS-T). Two hundred microliters of 1% PBS–bovine serum albumin (BSA) was used to block the plates for 1 h. After washing, plates were incubated with the 100 μL of respective standards and samples for 2 h, followed by a washing step. One hundred microliters of the respective detection antibody diluted in blocking solution was added to each well, and the plate was incubated for 1 h. After washing, the plate was incubated with 100 μL of avidin-horseradish peroxidase (HRP) conjugate (Biolegend) diluted in 1% PBS–BSA for 30 min. After washing, 100 μL of TMB (3,3′,5,5′-tetramethylbenzidine) substrate (Biolegend) was added to each well. The reaction was stopped by adding 100 μL of stop solution (1 M H_3_PO_4_). Optical density was read at 450 nm with a microplate reader (SPECTROstar Nano).

### Transfection.

HT29 cells were transiently transfected with EGFP fusion plasmids expressing either EGFP, EGFP-RIPK2, or EGFP-RIPK2 Y474F. Transfection into HT29 cells was carried out using Lipofectamine 3000 transfection reagent (Invitrogen; catalog no. L3000001) in Opti-MEM reduced serum medium (Gibco; catalog no. 31985062). A transfection mixture containing 1 μg EGFP fusion plasmid, Lipofectamine 3000 reagent, and P3000 reagent was prepared according to the manufacturer’s protocol. A total of 60,000 cells per well were cultivated together with the transfection mixture for 72 h at 37°C in a CO_2_ incubator. Cells were then treated with ETEC WT OMVs (final concentration of 100 ng/mL protein equivalent) or saline as a solvent control and incubated for additional 10 h. Transfected cells were then subjected to microscopic analyses.

### Microscopy.

Live-cell imaging was performed using a Leica SP8 confocal microscope with spectral detection and an HC FLUOTAR L 25×/0.95 VISIR water immersion objective. Dead cells or cells with compromised plasma membranes were stained by adding 200 μL of propidium iodide (PI)-RNase staining solution (200 μL PI solution to 300 μL of serum free cell culture medium) (Cell Signaling, Inc.; catalog no. 4087S) for 5 min at room temperature prior to imaging. PI labels only cells with compromised plasma membranes and dead cells. Such cells were excluded from the analysis. PI was excited at 561 nm, and emission was detected between 600 and 700 nm. GFP was excited at 488 nm, and emission was detected between 500 and 550 nm. Fluorescence and transmission images were acquired simultaneously. Maximum-intensity projections of acquired z-stacks (sampling of 0.08 by 0.08 by 5 μm) were generated using the open-source software Fiji (96).

### Knockdown.

RIPK2-specific siRNA RIP_8 (Qiagen Flexitube 1027415; catalog no. SI02758819) and a nontargeting ALLStars negative-control siRNA (catalog no. 1027280) were ordered from Qiagen. Transfection into HT29 cells was carried out essentially as described in the “Transfection” section with Opti-MEM reduced serum medium (Gibco; catalog no. 31985062) using Lipofectamine 3000 transfection reagent (Invitrogen; catalog no. L3000001), P3000 reagent, and 20 nM siRNA RIP_8 or the ALLStars negative-control siRNA. RIPK2 knockdown was performed for 72 h, followed by addition of ETEC WT OMVs (final concentration of 100 ng/mL protein equivalent) or an equivalent volume of saline as a solvent control. After an additional 16 h, cell supernatant and cell lysate were harvested and subjected to cytokine ELISA and immunoblot analyses, respectively.

### Immunoblot analyses.

Cell lysates were harvested from siRNA-treated HT29 cells after treatment with or without ETEC WT OMVs (final concentration of 100 ng/mL protein equivalent). Cells were briefly rinsed with PBS and lysed with ice-cold radioimmunoprecipitation assay (RIPA) buffer (50 mM Tris HCl, 150 mM NaCl, 1.0% [vol/vol] NP-40, 0.5% [wt/vol], sodium deoxycholate, 1.0 mM EDTA, 0.1% [wt/vol] SDS, 0.01% [wt/vol] sodium azide [pH 7.4]). The protein concentration in the lysate was determined using the Bradford assay (with Bio-Rad Laboratories protein assay dye reagent) according to the manufacturer’s manual. Lysates were separated by SDS-PAGE using 12% gels and the PageRuler prestained protein ladder (Thermo Fisher Scientific) as a molecular mass standard. Ten micrograms of each sample was loaded and transferred to a nitrocellulose membrane (Amersham) for immunoblot analyses, which were essentially performed as described previously (97). Anti-RIP2 antibody (abcam, ab8428) and glyceraldehyde-3-phosphate dehydrogenase (GAPDH) (14C10) antibody (Cell Signaling; 2118S) were used in combination with the HRP-conjugated anti-rabbit IgG (Jackson ImmunoResearch; 111-035-003) as the primary and secondary antibodies, respectively.

### Uptake assay.

Cellular uptake assays using octadecyl rhodamine B chloride (R18)-labeled OMVs were performed as described previously (14, 38, 98). R18-labeled OMVs were diluted in cell culture medium depleted of FCS to a final concentration of 10 ng/μL based on quantification by Bradford assay. In the case of proteinase K treatment, MVs were incubated overnight at 55°C with 100 μg/mL proteinase K, followed by inactivation for 10 min at 65°C prior to the addition in cell culture. HT-29 or Caco-2 cells were seeded into black 96-well plates and incubated for 24 h at 37°C. Cells were washed with 200 μL medium depleted of FCS, and 1 μg of protein equivalent of MVs was added per well. In case of addition of uptake inhibitors, commercially available agents were added at the following concentrations: wortmannin, 0.1 μM in DMSO (Sigma-Aldrich); cytochalasin D, 0.5 μM in DMSO (Sigma-Aldrich); chlorpromazine, 0.35 μM in ddH_2_O (Sigma-Aldrich); amiloride, 0.1 mM in DMSO (Sigma-Aldrich); nystatin, 0.2 μM in DMSO; (Sigma-Aldrich); and dynasore, 80 μM in ddH_2_O (Sigma-Aldrich). Cells were incubated at 37°C, and fluorescence was measured for 8 h.

### Statistical analysis.

Unless stated otherwise the data are presented as median with interquartile range (IQR). Statistical differences between data sets were analyzed by a Mann-Whitney U test for single comparison or a Kruskal-Wallis test, followed by Dunn’s *post hoc* test in case of multiple comparisons. Differences were considered significant for *P* values of <0.05.
