# Supplementary material for: Characterization of the Inflammatory Response Evoked by Bacterial Membrane Vesicles in Intestinal Cells Reveals an RIPK2-Dependent Activation by Enterotoxigenic Escherichia coli Vesicles
Source: Microbiol Spectr. 2023 Jun 12;11(4):e01115-23. doi: 10.1128/spectrum.01115-23 (PMC10433812; doi:10.1128/spectrum.01115-23)
Supplement: Supplemental file 1 — Fig. S1 to S6 and Tables S1 to S4. Download spectrum.01115-23-s0001.pdf, PDF file, 2.3 MB [file spectrum.01115-23-s0001.pdf]

Supplemental Figures and Legends

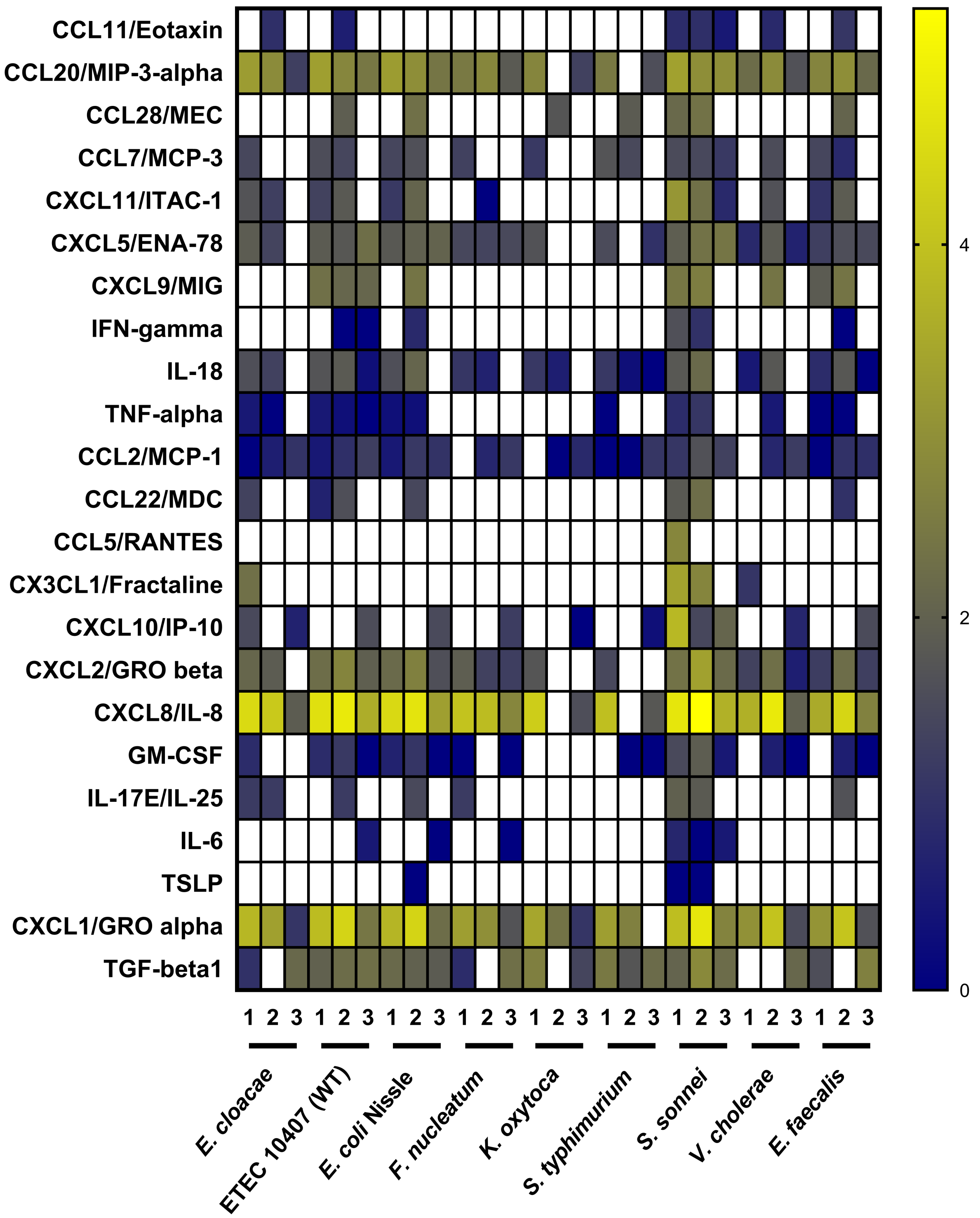

Figure S1. Heat map showing the induction of cytokine release by intestinal epithelial cells exposed to different bacterial MVs. Cytokine levels were quantified by Luminex analyses from supernatants of intestinal epithelial cells exposed for 16 h to different bacterial MVs (n = 3). Individual cytokines are indicated on the left. Intestinal epithelial cells [HT-29 (1), HT-29 MTX (2) and Caco-2 (3)] and MV donor strains are indicated on the bottom. Incubation with

saline (no MVs) served as mock-treated control to determine the non-stimulated secretion level for each cytokine in the respective cell line, which was subtracted as blank from MV exposed samples. Finally, values were log10 transformed. Blue boxes show low levels and yellow boxes show high levels of cytokine release upon MV exposure (see scale). White boxes indicate no measurable induction of cytokine release compared to the mock-treated controls.

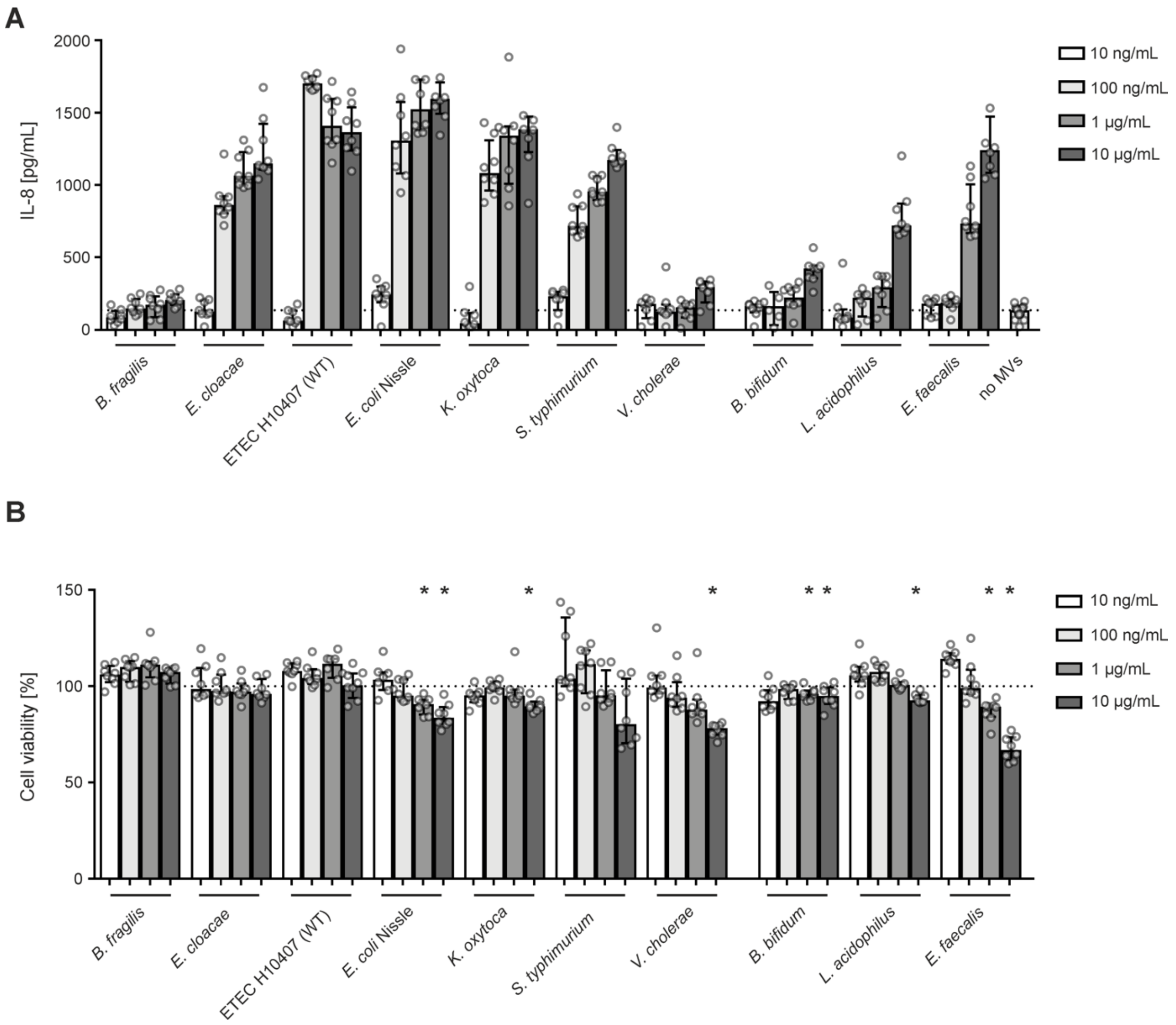

Figure S2: IL-8 response and cell viability of HT-29 intestinal cells after incubation with different amounts of MVs derived from diverse intestinal bacteria. (A) Cytokine levels were quantified by ELISA in supernatants of HT-29 intestinal cells incubated for 16 h with different amounts of MVs indicated by the gray scale. Donor strains of the MVs are indicated on the x-axis, respectively. Cells incubated with saline served as controls. Data is indicated as median ± interquartile range (n = 16 for no MVs and n = 8 for all other data sets). **(B)** Shown is the cell viability of HT-29 intestinal cells determined by MTT assays. Cells were incubated for 16 h with different amounts of MVs as indicated by the gray scale. Donor strains of the MVs are indicated on the x-axis, respectively. Cells incubated with saline served as controls and were set to 100% viability. Data is indicated as median ± interquartile range (n = 8). Asterisks highlight data sets with significantly reduced cell viability (* $P < 0.05$, Wilcoxon signed rank test against a hypothetical value of 100).

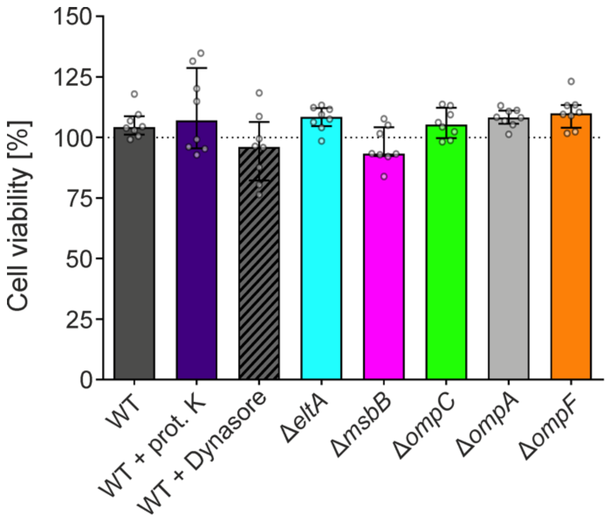

Figure S3: Cell viability is not affected upon exposure with OMVs from ETEC WT or mutants. Shown is the cell viability of HT-29 intestinal cells determined by MTT assays. Cells were exposed to OMVs (100 ng protein equivalent) from ETEC WT or mutants. Donor strains of the OMVs are indicated on the x-axis, respectively. Cells incubated with saline served as controls and were set to 100% viability. Data is indicated as median ± interquartile range (n = 8). None of the data sets showed a significantly reduced cell viability (* $P < 0.05$, Wilcoxon signed rank test against a hypothetical value of 100).

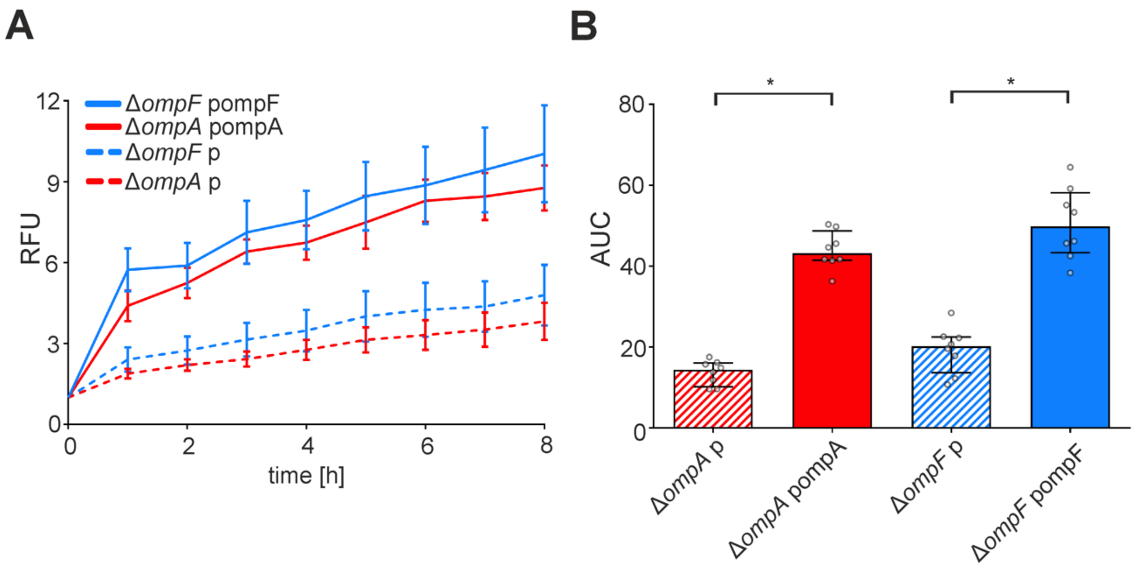

Figure S4: Expression of OmpA and OmpF in trans restores MV uptake in intestinal epithelial cells. (A) HT-29 intestinal cells were incubated for 8 h with rhodamine-labeled MVs derived from ETEC^{H10407} Δ*ompF* p, Δ*ompA* p, Δ*ompF* pompF and Δ*ompA* pompA. Uptake is detected by an increase in relative fluorescence units (RFU) measured every hour. Wells containing rhodamine-labeled MVs without cells served as a blank. Shown is the mean ± SD, n = 8. **(B)** Shown are the median area under the curve (AUC) values ± interquartile range retrieved from the uptake analyses presented in panel A. Asterisks highlight significant differences between respective data sets (* $P < 0.05$ Kruskal–Wallis test followed by Dunn's post hoc test).

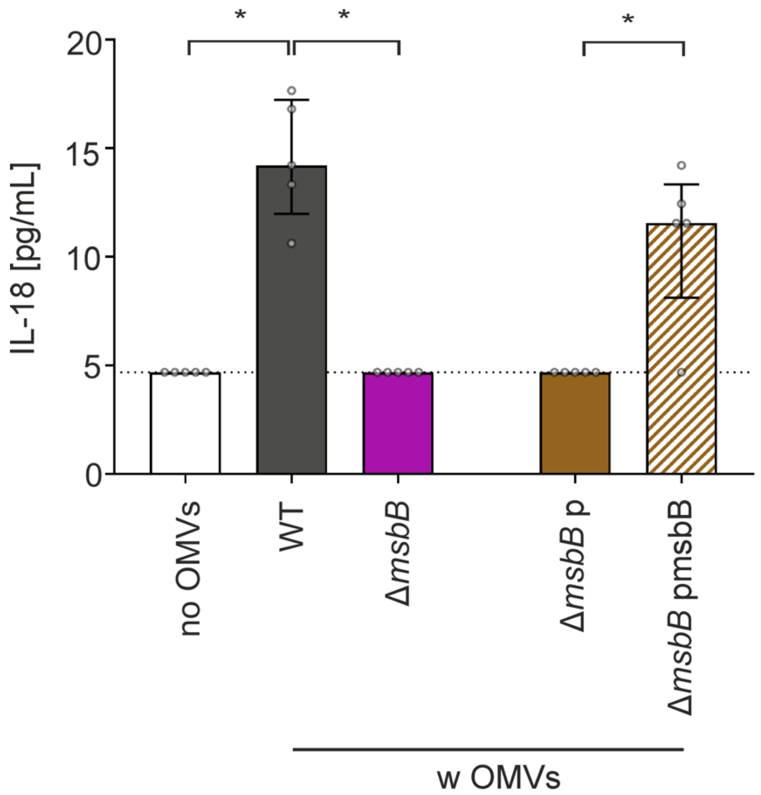

Figure S5: IL-18 response in HT-29 intestinal cells to MVs derived from ETEC^{H10407}. IL-18 levels were quantified by Luminex analyses in supernatants of HT-29 intestinal cells incubated for 16 h with MVs (100 ng/ml protein equivalent determined by Bradford). Donor strains of the MVs, presence of the caspase-1, -4 & -5 inhibitor (Z-YVAD-FMK) or the appropriate solvent control (DMSO) are indicated on the x-axis, respectively. Incubation with saline (no MVs) served as negative control. For samples without detectable levels of IL-18, the value was set to the limit of detection of 4.68 pg/mL (dotted line). Data is indicated as median ± interquartile range (n = 5). Asterisks highlight significant differences between respective data sets (* $P < 0.05$, Kruskal–Wallis test followed by Dunn's post hoc test for multiple comparisons or Mann Whitney U-test for single comparisons).

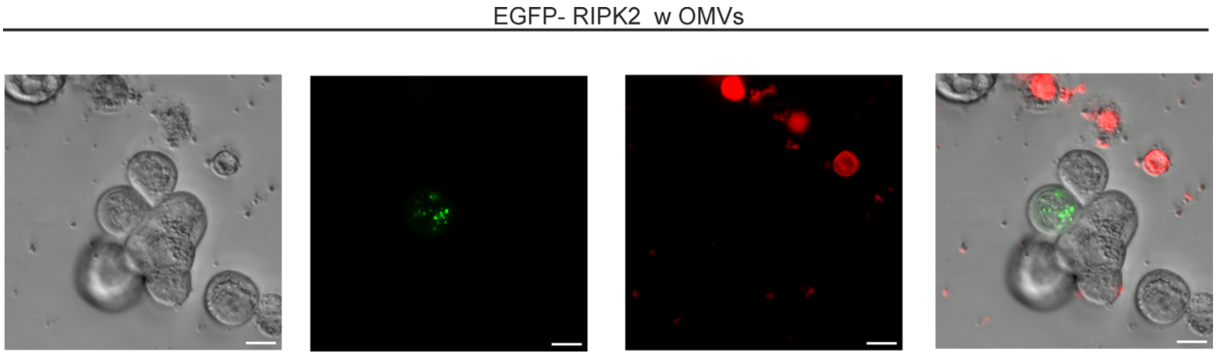

Figure S6: Representative image for identification of dead cells. Shown are examples of HT-29 cells transfected with an expression plasmid for EGFP-RIPK2**.** Control propidium iodine (PI) staining allowed visualization of dead cells (red), which were excluded from further analysis. RIPosome formation (green dots) of EGFP-RIPK2 upon exposure with MVs was only analyzed for viable cells (no red fluorescence signal). Scale bar = 10 µm.

Supplemental Tables

Table S1. Biomass quantification of OMVs derived from ETEC WT and mutants used in this study. Biomass quantification of OMV preparations derived from the respective cultures were analyzed for protein (determined by Bradford assays) or lipopolysaccharide (determined by Purpald assay). The ratio was calculated by dividing the respective protein biomass by the LPS biomass of each OMV preparation. Mean with standard deviation are shown (n = 4).

OMV donor	protein biomass by Bradford [µg/µL]	LPS biomass by Purpald [µg/µL]	ratio [protein/LPS]
WT	4.09 ± 0.57	0.05 ± 0.00	83.51 ± 5.38
Δ*eltA*	3.31 ± 1.32	0.10 ± 0.09	65.53 ± 39.08
Δ*msbB*	2.20 ± 0.07	0.06 ± 0.02	38.37 ± 14.14
Δ*ompC*	1.40 ± 0.34	0.09 ± 0.01	15.98 ± 4.53
Δ*ompA*	6.50 ± 0.86	0.23 ± 0.02	28.88 ± 4.62
Δ*ompF*	3.03 ± 0.21	0.16 ± 0.02	19.45 ± 1.62

Table S2. Strains and plasmids used in this study.

bacterial strains	description	reference
DH5αλpir	*Escherichia coli*, F^- *endA1 glnV44 thi-1 recA1 relA1 gyrA96 deoR nupG* Φ80d*lacZ*ΔM15 Δ(*lacZYA-argF*) U169 *hsdR17*(r_K^- m_K^+) λ*pir*RK6	(80)
SM10λpir	*E. coli, thi thr leu tonA lacY supE recA::*RPA-2-Te::Mu λ*pir*, Km^R	(80)
WT	Wild type enterotoxigenic *E. coli* (ETEC), spontaneous Sm^r mutant of H10407, serotype O78:H11, CFA/I LT+ STh+ STp+, Sm^R	(81)
Δ*ompA*	Deletion of *ompA* in WT, Sm^R	This paper
Δ*ompF*	Deletion of *ompF* in WT, Sm^R	This paper
Δ*msbB*	Deletion of *msbB* in WT, Sm^R	(25)
Δ*eltAB*	Deletion of *eltAB* in WT, Sm^R	This paper
Δ*ompC*	Deletion of *ompC* in WT, Sm^R	This paper
A. muciniphila	*Akkermansia muciniphila*, clinical isolate from the Medical University Graz, anaerobic cultivation	(82)
B. fragilis	*Bacteroides fragilis*, ATCC® 25285, NCTC 9343, anaerobic cultivation	
B. thetaiotaomicron	*Bacteroides thetaiotaomicron*, ATCC® 29148, anaerobic cultivation	
B. vulgatus	*Bacteroides vulgatus*, human isolate, strain collection Medical University of Graz, anaerobic cultivation	Institute of Hygiene, Medical University of Graz
C. difficile	*Clostridium difficile*, strain collection Medical University of Graz, DSM 1296, anaerobic cultivation	Institute of Hygiene, Medical University of Graz
B. bifidum	*Bifidobacterium bifidum, ATCC*® 29521, anaerobic cultivation	
B. longum	*Bifidobacterium longum* BB536, clinical isolate from healthy human infant, 1969, anaerobic cultivation	(83)
E. cloacae	*Enterobacter cloacae* human isolate, strain collection Medical University of Graz	Institute of Hygiene, Medical University of Graz
E. faecalis	*Enterococcus faecalis*, strain collection Medical University of Graz, ATCC® 29212, DSM 2570	Institute of Hygiene, Medical University of Graz
EAEC 042	enteroaggregative *E. coli*, O44:H18 042, clinical isolate from Lima, Peru, 1983	Institute of Hygiene, Muenster
EAEC 17-2	enteroaggregative *E. coli*, O3:H2, clinical isolate from Chile	Institute of Hygiene, Muenster
EAEC 55989	enteroaggregative *E. coli*, clinical isolate from an HIV patient in Central African Republic, 2002	Institute of Hygiene, Muenster
EIEC EDL 1284	enteroinvasive *E. coli*, O124:H^-, strain collection Institute of Hygiene, Muenster	Institute of Hygiene, Muenster
EIEC HN280	enteroinvasive *E. coli,* O135, strain collection Institute of Hygiene, Muenster	Institute of Hygiene, Muenster
ETEC E1392-75	enterotoxigenic *E. coli*, O6:H16, strain collection Institute of Hygiene, Muenster	Institute of Hygiene, Muenster

EPEC E2348/69	enteropathogenic *E. coli*, O127:H6, strain collection Institute of Hygiene, Muenster	Institute of Hygiene, Muenster
UPEC CFT073	uropathogenic *E. coli*, O6:K2:H1, strain collection Institute of Hygiene, Muenster	Institute of Hygiene, Muenster
UPEC 536	uropathogenic *E. coli,* O6:K15:H31, strain collection Institute of Hygiene, Muenster	Institute of Hygiene, Muenster
UPEC UTI89	uropathogenic *E. coli*, O18:K1:H7, strain collection Institute of Hygiene, Muenster	Institute of Hygiene, Muenster
E. coli Nissle	*E. coli* strain Nissle 1917	(51)
F. nucleatum	*Fusobacterium nucleatum*, clinical isolate from the Medical University Graz, anaerobic cultivation	(84)
K. oxytoca	*Klebsiella oxytoca* AHC-6, clinical isolate from AAHC patient (acute phase)	(85)
K. pneumonia	*Klebsiella pneumonia* C3091, UTI isolate	(86)
L. acidophilus	*Lactobacillus acidophilus*, ATCC 4356, DSM 20079, anaerobic cultivation	(87)
P. acidilactici	*Pediococcus acidilactici*, human isolate, strain collection Medical University of Graz, anaerobic cultivation	Institute of Hygiene, Medical University of Graz
P. vulgaris	*Proteus vulgaris*, human isolate, strain collection Medical University of Graz	Institute of Hygiene, Medical University of Graz
S. typhimurium	*Salmonella enterica* serovar Typhimurium, strain 14028s, Institute of Hygiene, Muenster	Institute of Hygiene, Muenster
S. flexneri	*Shigella flexneri*, human isolate, strain collection Medical University of Graz	Institute of Hygiene, Medical University of Graz
S. sonnei	*Shigella sonnei*, human isolate, strain collection Medical University of Graz	Institute of Hygiene, Medical University of Graz
V. cholerae	*Vibrio cholerae*, spontaneous Sm^R mutant of E7946, clinical isolate from Bahrain 1978, serogroup O1, biotype El Tor, serotype Ogawa, Sm^R	(88)
Y. enterocolitica	*Yersinia enterocolitica,* clinical isolate, JB580v serotype strain 8081, serotype O:8	(89)
**plasmids**		
pCVD442	*ori6K mobRP4 sacB*, Ap^R	(73)
pCVDΔompA	pCVD442 with up- and downstream fragments of *ompA*, Ap^R	This paper
pCVDΔompF	pCVD442 with up- and downstream fragments of *ompF*, Ap^R	This paper
pCVDΔompC	pCVD442 with up- and downstream fragments of *ompC*, Ap^R	This paper
pCVDΔeltAB	pCVD442 with up- and downstream fragments of *eltAB*, Ap^R	(25)
p	pBAD18-Kan Expression vector, arabinose inducible, Kan^r	(90)
pompA	Expression plasmid with *ompA* of H10407 including a C-terminal HIS-tag in pBAD18-Kan, Kan^r	This paper

pompF	Expression plasmid with *ompF* of H10407 including a C-terminal HIS-tag in pBAD18-Kan, Kan^r	This paper
pmsbB	Expression plasmid with *msbB* of H10407 in pBAD18-Kan, Kan^r	This paper
pEGFP	Expression plasmid pcDNA5/FRT/TO-EGFP allowing expression of EGFP, Ap^R	(45)
pEGFP-RIPK2	Expression plasmid pcDNA5/FRT/TO-EGFP-RIPK2 allowing expression of EGFP-RIPK2, Ap^R	(45)
pEGFP-RIPK2 Y474F	Expression plasmid pcDNA5/FRT/TO-EGFP-RIPK2 Y474F allowing expression of EGFP-RIPK2 Y474F, Ap^R	(45)

Table S3. Oligonucleotides used in this study.

**Oligonucleotides**		
ompA_SacI_1	AAAGAGCTCCGTGTCGTCAACGGTCAGG	This paper
ompA _EcoRI_2	TGAATTCTTTTTGCGCCTCGTTATCATC	This paper
ompA _EcoRI_3	TTTGAATTCGTTCTCGTCTGGTAGAAAAAC	This paper
ompA _XbaI_4	AAATCTAGACAGCAGTGTACGCAAAGAGA	This paper
ompC_SacI_1	AAAGAGCTCCTGTTATCCTGACCATTCAGG	This paper
ompC _EcoRI_2	TTAGAATTCGTTATTAACCCTCTGTTATATG	This paper
ompC _EcoRI_3	TTTGAATTCTCTCGATTGATATCGAACAAAG	This paper
ompC _XbaI_4	AAATCTAGACACCATCACGCAAAAACCAAC	This paper
ompF_SacI_1	TTTGAGCTCCATGCTGAAATATGTCTTCAAAG	This paper
ompF_EcoRI_2	AAAGAATTCCATCATTATTTATTACCCTCATG	This paper
ompF_EcoRI_3	TTTGAATTCTAGCACACCTCTTTGTTAAATG	This paper
ompF_XbaI_4	AAATCTAGAGGTTTGCGCCTTTTTCCTGC	This paper
ompA_SacI_fw	AATGAGCTCGGTGAAGGATTTAACCGTGTTATCTCGTTGGAGATATTCATGG TGTAT	This paper
ompA_HIS_XbaI_rev	AAATCTAGACTAGTGGTGATGGTGATGATGAGCCTGCGGCTGAGTTACAACG TCTTTGATACC	This paper
ompF_SacI_fw	AAAGAGCTCTTGACGGCAGTGGCAGG	This paper
ompF_HIS_XbaI_rev	ATATCTAGACTAGTGGTGATGGTGATGATGGAACTGGTAAACGATACCCAC AGC	This paper
msbB_EcoRI_fw	CACGAATTCAACTGGAAAAGCATGGAAACGAA	This paper
msbB_XbaI_rev	CACTCTAGATTATTTGATGGGATAAAGATCTTTGCG	This paper

[1] restriction sites are underlined

Table S4. Protein biomass quantification without and with SDS of representative MVs used in this study. Biomass quantification of four MV preparations derived from different donors as indicated were analyzed for their protein amount (determined by Bradford assay) without (w/o) and with (w) lysis using 0.1 % SDS. Fold increase upon lysis was calculated by dividing the respective protein biomass obtained with SDS by protein biomass obtained without SDS. Individual values and mean with standard deviation are shown (n = 4).

MV donor	MV Protein concentration w/o SDS [µg/µL]	MV Protein concentration w SDS [µg/µL]	fold increase [w SDS/ w/o SDS]	Average fold increase (Mean ± SD)
B. fragilis	2.94	4.29	1.46	1.38 ± 0.07
	3.36	4.33	1.29	
	3.25	4.34	1.34	
	2.84	4.13	1.45	
E. cloacae	2.59	3.45	1.33	1.28 ± 0.07
	2.63	3.55	1.35	
	2.64	3.29	1.25	
	2.60	3.07	1.18	
ETEC H1047 (WT)	3.81	4.75	1.25	1.43 ± 0.12
	4.58	6.38	1.41	
	4.68	7.33	1.57	
	3.28	4.95	1.51	
E. coli Nissle	0.93	1.34	1.45	1.44 ± 0.03
	0.98	1.43	1.46	
	0.90	1.25	1.38	
	0.84	1.22	1.45	
K. oxytoca	2.00	2.58	1.29	1.38 ± 0.10
	2.40	3.66	1.52	
	2.40	3.09	1.29	
	4.33	6.14	1.42	
S. typhimurium	5.10	7.52	1.47	1.45 ± 0.03
	5.33	7.48	1.40	
	4.83	7.04	1.46	
	5.40	7.93	1.47	
V. cholerae	3.40	4.66	1.37	1.44 ± 0.14
	3.80	4.76	1.25	
	2.60	4.01	1.54	
	2.82	4.52	1.60	
B. bifidum	1.44	1.86	1.30	1.38 ± 0.13
	1.17	1.86	1.59	
	0.74	1.03	1.39	
	1.58	1.95	1.24	
L. acidophilus	0.54	0.76	1.41	1.38 ± 0.11
	0.31	0.45	1.43	
	0.30	0.36	1.20	
	0.21	0.31	1.49	
E. faecalis	2.48	3.60	1.45	1.40 ± 0.13
	0.39	0.53	1.38	
	0.48	0.58	1.20	
	0.36	0.55	1.55	
